# Construction and demolition waste recycling in developing cities: management and cost analysis

**DOI:** 10.1007/s11356-022-23502-x

**Published:** 2022-11-07

**Authors:** Navarro Ferronato, Rocio Clara Fuentes Sirpa, Edith Gabriela Guisbert Lizarazu, Fabio Conti, Vincenzo Torretta

**Affiliations:** 1 Department of Theoretical and Applied Sciences (DiSTA), University of Insubria, Via G.B. Vico 46, 21100 Varese, Italy; 2 Universidad Mayor de San Andrés (UMSA), IIDEPROQ, Calle 30, Cota Cota, La Paz, Bolivia

**Keywords:** Developing countries, Waste flow analysis, Circular economy, Latin America, Solid waste management, Sustainable development

## Abstract

In Bolivia, construction and demolition waste (CDW) is unmanaged. The current research implemented a CDW flow analysis and a cost assessment in the city of La Paz, aiming to compare future recycling scenarios. Lack of data is challenging; therefore, a review of 31 international studies has been conducted. Results report that the waste generation rate (WGR) of concrete residential buildings can be of about 91.9–113.3 kg m^−2^ for construction and about 867.2–1064.8 kg m^−2^ for demolition. In addition, positive correlation was found in national WGR with number of inhabitants and the GDP. By these results, it was estimated that about 271,051–349,418 tonnes of CDW are potentially generated yearly in La Paz, about two times more than the CDW projected by local authorities. By the scenarios analysis, it was estimated that 56.1–71.1% of the inert aggregates can be recycled. The cost of the system is estimated between 7.8 and 31.1 USD t_CDW_^−1^, for a total annual cost (20 years horizon) of about 2.3–9 million USD y^−1^ depending on the management scenario. In the best scenario, an average fee of less than 1% of the current costs of a flat in La Paz can be required, potentially affordable for the local economy. The most attractive scenario involves the source separation of recyclable materials and the hybrid organization of stationary and mobile recycling facilities. Results show that a CDW management can be implemented in La Paz to foster a circular economy in developing cities.

**Supplementary Information:**

The online version contains supplementary material available at 10.1007/s11356-022-23502-x.

## Introduction

In Bolivia, construction and demolition waste (CDW) is disposed of in open dumping areas (Ferronato et al. 2021a). This is a typical issue in the whole Latin American region: in Brazil, performance indicators in relation to CDW management are well below those found in developed countries, although waste open dumping is banned since more than 15 years (Nunes and Mahler 2020); in Perú, there is no legislation that encourages the use of recycled concrete and final disposal is most of the time informal (Rondinel-Oviedo 2021). In México, significant amount of CDW is discarded in clandestine dumpsites, while controlled disposal sites lack appropriate engineering measures (Araiza-Aguilar et al. 2019). Actions towards the implementation of appropriate CDW management systems in Latin America are urgent.

CDW mismanagement is due to various factors, such as population growth, urbanization, gross domestic product (GDP), and CDW management regulatory measures (Aslam et al. 2020). In addition, the lack of data and reliable information is also affecting the transition towards a circular approach. This is a typical issue in developing countries. For example, in Romania, uncontrolled CDW disposal is associated with the lack of collection and treatment facilities, the regional waste governance crisis, and the absence of markets for recyclable aggregates (Mihai 2019). In the Republic of Macedonia, CDW is mainly dumped in open areas, with more than 50 uncontrolled sites in the city of Skopje (Bianchini et al. 2020). In Saudi Arabia, subcontractors regularly dump the CDW informally, along roadsides and open areas, resulting in contamination, blocked roadways, and undesirable views (Blaisi 2019). Similarly, in Southeast Asia, lack of data, regulation, and monitoring of CDW management are resulting in waste uncontrolled disposal (Hoang et al. 2020). However, CDW recycling as secondary building materials is recommended (López Ruiz et al. 2020): the recycling and recovery rate of CDW waste varies across the world, from below 10% in China and India to above 90% in Japan (Duan et al. 2019). More efforts should be spent in order to reduce the depletion of abiotic resources and the recovery of recyclable aggregates.

The research presented in this article aims to quantify the amount and flows of the CDW generated in a developing city. As a case study, La Paz, a representative Bolivian city, has been considered. Here, lack of primary data is a real issue. In addition, the research would estimate the expenses required for managing the total amount of CDW. A material flow analysis (MFA) has been conducted with the support of secondary data collected by a literature review of the international scientific literature and the analysis of the Bolivian documentation. The cost analysis has been conducted with secondary data as well, also with the support of preliminary information available from a local pilot plant built in April 2021 in La Paz.

At international level, MFA and scenarios analysis are commonly used to assess waste generation and management (Guo and Huang 2019; Ferronato et al. 2020b). In China, a dynamic MFA was conducted for Beijing’s urban housing system, for estimating the amount of CDW generated at municipal level (Hu et al. 2010). In Brazil, an MFA was implemented to assess the flows of CDW from the residential building stock in the city of Rio de Janeiro per different building types (Condeixa et al. 2017). In Vietnam, MFA was employed to assess recycling rates, processing quantities of informal and formal recyclers (Lockrey et al. 2016). Similarly, in Austria, MFA was used to determine how CDW reduction, re-use, and recycling can contribute to reduce the demand of raw materials in Vienna (Lederer et al. 2020). However, at a global level, very low information is available in terms of CDW management costs for a whole CDW management system where recycling is prioritized, while in Latin America, there are not reliable information about CDW generation and flows. This information should be provided to policy-makers in order to increase awareness about the implementation of appropriate CDW management systems and to allocate financial resources.

The research introduced in this article contributes, first, to highlight the possible challenges in terms of data reliability in Latin American cities, and second, to evaluate the financial expenditures potentially required in developing countries in order to build an appropriate CDW waste management system. In addition, the research provides the first analysis for quantifying an average waste generation rate (WGR) obtained by the international scientific literature that can be employed by engineers interested in estimating CDW flows in developing countries. Therefore, the objective of the research is to provide a potential range of costs that a public management system should afford to manage CDW. This information is mostly absent in Latin American cities, and it is important to provide at least an estimation of this expenses to support decision-makers and public strategies for appropriate management of CDW. In addition, providing the potential amounts of waste that can be potentially recycled and its financial benefits can assist policy-makers in supporting the transition towards a circular economy.

## Methods

### Overview of the approach

**Fig. 1:**
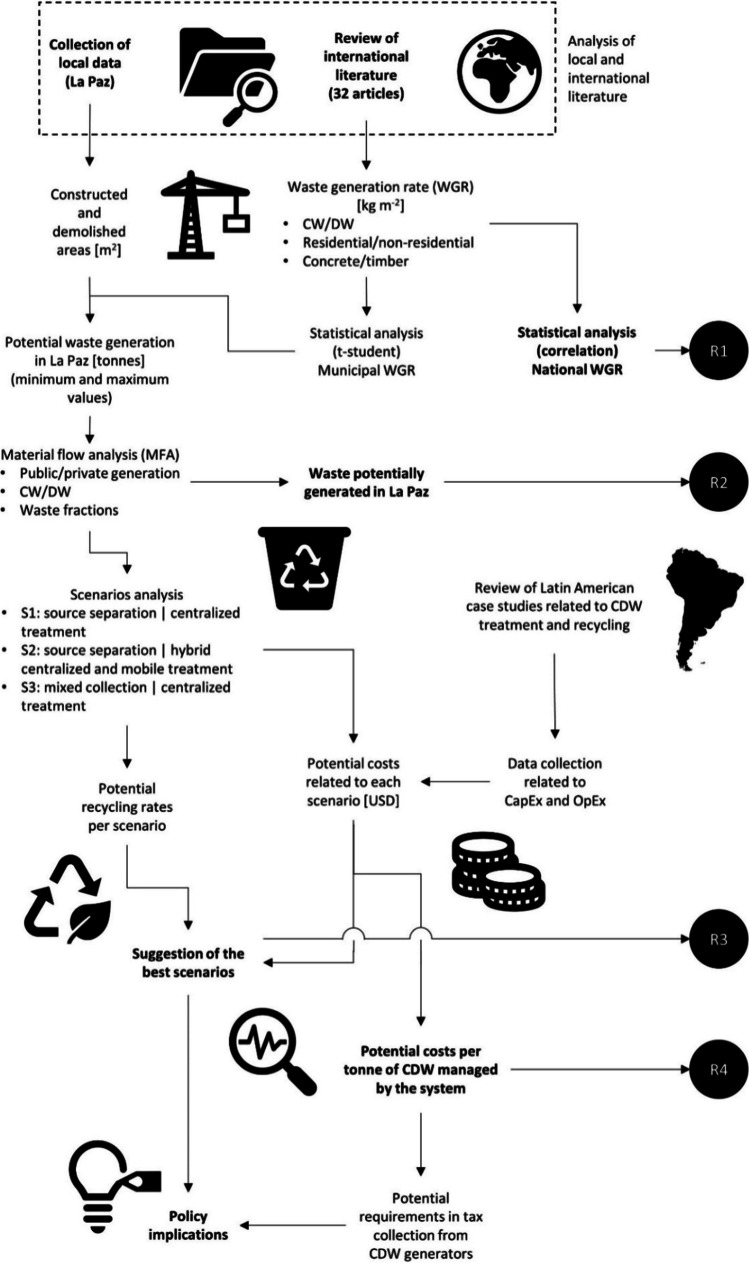
Scheme of the research procedure conducted in La Paz

In Fig. 1, the steps followed for the research are schematically presented. Overall, the study is carried out by the use of secondary data. The first step consists in collecting the local documentation, available from the municipal government, in order to estimate the areas demolished and constructed during the last 5 years (2013–2017). Then, a review of the international scientific literature has been conducted, where 31 articles and reports were selected for collecting information about the WGR and the waste characterization, both for construction and demolition activities. The information were gathered in function of the buildings size (residential or no residential) and the main construction materials (timber or concrete).

A statistical analysis has been carried out to include some data where no specifications were added. The scientific literature revised does not always provide indication about the source of the CDW, making difficult categorizing and grouping the WGR and waste characterization. In addition, a correlation analysis among the GDP and number of inhabitants allows evaluating their possible connection with the WGR (first result of the research—R1). The outcome of the literature review and documentation analysis allows calculating the potential CDW generation of La Paz (R2). The findings are presented in a range of values (minimum and maximum amounts) in order to consider the uncertainty due to secondary data.

The quantities of CDW estimated to be generated were employed for assessing the CDW flow of La Paz, mainly with two purposes: to quantify the waste fractions (recyclable aggregates, other recyclables, and others non-recyclable) possibly generated at municipal level and to compare recycling scenarios that can be implemented in La Paz for maximizing the recycling rate. Three scenarios were analyzed, function of the selected or mixed segregation, on-site or off-site recycling, and the combination of these variables. The assessment provides indication about the potential recycling rate that can be achieved by the best and worst management conditions (R3), as well as the potential yearly costs that the local government should afford (R4), both for operation (OpEx) and capitalization of goods and infrastructures (CapEx). The outcomes of the analysis give indications about the policy that should be considered for La Paz in the next decade of actions, as well as the potential taxes that should be introduced in order to cover the CDW management costs.

The case study introduced can be of reference for other similar developing cities. Implementing a statistical analysis of data reviewed gives a more robust evaluation of data that can be potentially used for estimating the WGR in other areas of the world with no data availability: this is the first research article that tried to do so. In addition, in other words, a meta-analysis has been performed to estimate potential range of values related to a WGR rather than simply reviewing the literature or rather than take only the old and limited info available in La Paz. The results obtained by the study can support the identification of quantities and costs of CDW management and generation in developing cities with limited data availability and can provide reliable range of values related to the case of La Paz.

### Study area

La Paz is a Bolivian developing city with about 950,000 inhabitants. The local municipal government is able to implement autonomous actions for the valorization and disposal of non-hazardous waste. In particular, recycling facilities, final disposal sites, recycling campaigns, and the establishment of waste fees should be planned and implemented by the local autonomous administration. However, as common in Bolivia, the solid waste management (SWM) systems are mainly subsidized by the national government, making difficult the implementation of new and innovative facilities. For this reason, CDW is still not formally managed.

Primary data about the CDW generated in La Paz are not available. Data are mainly available by secondary sources and indirect measures. In particular, the WGR for construction and demolition activities used at municipal level derived by a single analysis implemented before 2015, while the waste characterization is known only from the analysis of the waste disposed of in open dumping areas. It can be estimated that, at municipal level, in 2017, about 113,000 tonnes of CDW were generated, with more than 19 open dumping areas counted around the city (Ferronato et al. 2021a, b). The waste is transported by informal workers, paid by the waste generators, and the residues are discarded in open areas, such as rift, valleys, or rivers. This situation is typical in the whole country since no recycling policy of formal management has been implemented yet at national level. Similarly, specific laws and regulations are lacking at municipal and national scale. Infrastructures and know-how are completely lacking. Only pilot programs with the support of international donors were organized in the last years.

### Literature review

Data necessary for the estimation of CDW generation and flows have been collected and divided according to the generator (private or public), the source of generation (construction or demolition activities), the constructions’ size and type (residential, non-residential, and civil infrastructures), and the main materials used for buildings (timber or concrete with masonry). In particular, residential structures are high-rise buildings, skyscrapers, and houses with terraces, among others (Cochran et al. 2007), while non-residential are small-sized buildings like single-family houses or small shops. Finally, civil infrastructures are all other building activities like bridges, roads, and sewer systems.

#### Analysis of local documentation and interviews

Documentation available from the local municipal government has been reviewed. Through this documentation, which collect the registered information of the last 5 years, information about the typical construction typologies of La Paz and the municipal data of built and demolished areas has been collected. The documentation highlights the type of buildings of La Paz (houses, apartments, hospitals, hotels, etc.) and civil infrastructures (retaining wall, drains, vehicular bridges, etc.). Therefore, it was also possible to interpret the type of construction size (residential and non-residential) as well as the main material used for buildings (concrete with masonry). Therefore, data related to the constructed and demolished areas were extracted. These data are annual and are shown in a period between 2013 and 2017. Data were classified according to public and private buildings as well as constructed or demolished areas: the annual mean values and their respective standard deviation have been calculated.

Since the methodology used depends a lot on secondary data quality, at the end of 2020, unstructured interviews (face-to-face through virtual platforms) were carried out with municipal experts in CDW. These interviews were carried out after studying the municipal documentation, in order to resolve any uncertainty or need for information not available. The interviews confirmed that the documents revised were the most updated and reliable at municipal level, the main employed by local managers.

#### International scientific literature review

A literature review has been conducted for obtaining an average WGR and waste characterization. Articles, reports, and M.Sc. thesis published in the last 20 years, written in English or Spanish, and available in the Scopus databases were revised. These studies are published in scientific articles and proceedings belonging to journals like “Waste Management,” “Waste Management & Research,” and “Resources, Conservation and Recycling,” while technical reports or M.Sc. thesis refer to Latin America. Totally, 29 studies referring to the estimation and management of CDW from different cities were investigated. The list of publications is reported in Table 1, while the specific data collected by the analysis are reported in Table S1.

**Table 1:** List of references reviewed for data collection

n.**	Reference	City, country, or region	Inh. (millions)	GDP (USD inh^−1^)	System boundary	Methods for estimating the WGR and/or the CDW characterization	CDW generation	Structure (Concrete, timber)	Size (Residential, non-residential)
1	(Ram and Kalidindi 2017b)	Chennai, India	6.5	1680	Regional level	Secondary data: literature review	CW	N.A	N.A
						WGR assessed on site, within a case study (Primary data). Determination of constructed areas through a cash flow. Indirect determination of demolition waste through the WGR and the constructed areas	DW	Concrete	
								Masonry	
2	(Paz and Lafayette 2016)	Brazil	206.16	8920	Project level	N.A	CW	N.A	N.A
3	(Lu et al. 2011)	Shenzhen, China	11.91	3680	Project level	Primary data: estimation of CW quantity, areas and WGR from on-site measurements, through selected case studies	CW	Concrete	Residential
4	(Poon et al. 2004)	Hong Kong, China	6.78	1510	Project level	N.A	CW	N.A	N.A
5	(Kartam et al. 2004)	Kuwait	2.20	28,100	Regional level	N.A	CW	N.A	N.A
							DW	N.A	
6	(De Melo et al. 2011)	Lisbon, Portugal	0.55	22,720	Regional level	N.A	CW	N.A	N.A
7	(Bernardo et al. 2016)	Lisbon, Portugal	0.50	19,930	Project level and regional level	N.A	DW	N.A	N.A
8	(Bergsdal et al. 2007)	Norway	4.71	78,500	Regional level	N.A	CW	N.A	Residential
							DW	N.A	
9	(Al-Sari et al. 2012)	Palestine	11.60	NA	Project level	N.A	CW	N.A	N.A
10	(Coelho and De Brito 2011)	Portugal	10.56	22,720	Project level and regional level	N.A	CW	N.A	N.A
							DW	N.A	
11	(Ding and Xiao 2014)	Shanghai, China	23.02	7510	Regional level	N.A	CW	N.A	N.A
							DW	N.A	
12	(Li et al. 2013)	Shenzhen, China	11.91	6770	Project level	N.A	CW	N.A	N.A
13	(EPA 2009)	USA	290.11	39,770	Regional level	Combined methodology: determination from case studies with on-site measurements, reports, and research of national statistical data. Estimation of the amount of DW through calculation based on the area	CW	N.A	Residential and no residential
							DW	N.A	
14	(Sáez et al. 2014)	Spain	46.48	29,130	Project level	N.A	CW	N.A	N.A
15	(Solís-Guzmán et al. 2009)	Spain	46.36	32,460	Project level	N.A	CW	N.A	N.A
							DW	N.A	
16	(Llatas 2011)	Spain	46.74	30,950	Project level	N.A	CW	N.A	N.A
17	(Srour et al. 2013)	Beirut, Lebanon	6.01	7560	Project level and regional level	Combined methodology: determination of the areas from case studies with on-site measurements, interviews with contractors and data on fees charged. Estimation of the amount of DW through calculation based on the area	DW	Concrete	N.A
18	(Mañá et al. 2000)	Spain	40.57	15,770	Project level	N.A	CW	N.A	Residential and no residential
							DW	N.A	No residential
20	(Myhre 2000)	Norway	4.49	36,570	N.A	N.A	CW	N.A	No residential
21	(Ortiz et al. 2010)	Spain	46.58	31,880	N.A	N.A	CW	N.A	No residential
22	(Cochran et al. 2007)	Florida, USA	18.26	48,500	N.A	N.A	CW	N.A	No residential
							DW	N.A	
23	(Martínez Lage et al. 2010)	Galicia, Spain	2.77	31,880	Project level and regional level	N.A	CW	N.A	Residential
							DW	N.A	
24	(Vancouver 2008)	Canada	33.25	34,800	N.A	N.A	DW	N.A	Residential
25*	(Mália et al. 2013)	General	-	-	Regional level	Indirect methodology: determination of WGR from bibliographic research	CW	Concrete	Residential
								Timber	
								Masonry	
								N.A	
							DW	Concrete	
								Timber	
								Masonry	
								N.A	
26	(Mah et al. 2016)	Malaysia	30.27	10,680	Project level	Direct methodology: estimation of quantity of CDW, areas and WGR from measurements of truck records and site visits	CW	Timber	Residential
								Mixed	
							DW	N.A	
27	(Umar et al. 2018)	Malaysia	30.27	10,680	Regional level	Indirect methodology: determination of WGR from bibliographic research. Building number data through local research. Estimation of amount of CW through calculation based on the area	CW	N.A	Residential
28	(Mercader-Moyano and Ramírez-De-Arellano-Agudo 2013)	Sevilla, Spain	1.93	29,520	Regional level and case study	Direct methodology: estimation of CW, areas and WGR from on-site measurements, through selected case studies	CW	Concrete	Residential
29	(Kofoworola and Gheewala 2009)	Thailand	64.20	2790	Regional level	Indirect methodology: data from bibliographic research. Determination of areas through local investigation for building licenses. Estimation of amount of CW through calculation based on the area	CW	N.A	Residential and No Residential

*Reference n. 25 does not involve a specific geographic region since it is a compilation of several international studies. For this reason, it is classified as “general,” and no information is given on the number of inhabitants or economic level

**Numbers reported in function of author’s needs. N. 19 is missing since only qualitative information were collected

*N.A* Not available

*CW* construction waste, *DW* demolition waste

Data obtained from the international bibliography have two types of physical units: mass and volumetric. Therefore, to standardize the data to just one unit, material density data has been compiled through international literature. Mass units were used for the study since volumes can change due to compaction or other processing (EPA 2009). Average density values have been collected from the scientific literature (Mália et al. 2013; Srour et al. 2013; Mah et al. 2016; Ram and Kalidindi 2017a). The information collected is reported in Table S2.

After data collection, determination of mean values and standard deviation were carried out. The WGR and waste characterization were not always specifically associated to structures’ class and size (residential, and non-residential) or to a building material (concrete or timber). Therefore, statistical analysis between groups of data was implemented in order to add or remove data from a group to another. The aim was to allocate the maximum number of data collected from the international scientific literature to a specific class. In addition, for the research, CDW characterization has been grouped in six categories: inert waste (concrete, bricks, masonry, mortar, sand, and soil), wood and timber, metals, gypsum, hazardous waste, and others (plastics, glass, paper, and cardboard).

#### Statistics

Data collection is heterogeny in nature. Studies collected by the scientific literature do not always provide indications about the origin of the CDW, the methods employed for the analysis, or the type of structure where the waste was generated. Therefore, a statistical analysis was carried out with the intention of knowing whether or not there was a statistically difference between each group of WGR and waste characterization. In particular, statistics were made in order to consider data where specifications about the origin were not available.

Two hypotheses were performed: WGR are different in function of different construction types. Two parameters are considered: the construction size (residential, non-residential, and unknown); and the construction structure (concrete with masonry, timber, and unknown); CDW characterization is different from dissimilar generation activities. Therefore, as for the WGR, the two parameters that may influence waste composition are considered for the analysis.

Statistical analysis was not carried between construction waste (CW) and demolition waste (DW) since the international literature accepts a statistically difference between their values (EPA 2009). A *t*-test has been conducted, with a confidence level of 95% (*p* < 0.05). RStudio® has been used for the analysis. When statistically different, the groups were not merged, and data where construction size or structure are not specified were discarded. On the other hand, when no statistical difference has been found, all data were grouped and used for the analysis. The mean value and the standard deviation obtained are used as reference values to estimate the potential CW and DW that can be generated in La Paz.

In parallel, a correlation analysis has been conducted. The aim is to find additional information, thanks to the literature review implemented. The number of inhabitants and GDP were correlated with the WGR both for CW and DW that were estimated at national level. In addition, linear regression has been considered in order to present the results in case of significant correlation. A *p* value equal or lower to 0.05 has been considered significant.

### Waste flow analysis

The indirect methodology focuses on the quantitative estimation of CDW from an area-based calculation. From the data obtained by the literature review, documentation analysis, and statistical tests, CW and DW generation were estimated. The product between the constructed or demolished areas and the specific average WGR allows obtaining the potential amount of waste that can be obtained by the system. In addition, the availability of the standard deviation for each value allows considering uncertainty of data. Uncertainty has been obtained through the product function corresponding to the propagation of errors. Therefore, the standard deviation of the amounts of CDW potentially generated annually in the municipality of La Paz is used by the analysis. To obtain the CDW flow, the STAN® software has been used as a calculation tool (Cencic 2016).

The MFA has been used specifically to assess the flows of generation activities: (i) CW and DW, based on the estimated results of total amounts of CDW of each type of construction; (ii) estimation of private and public CDW generation; and (iii) material flows available within the CDW, based on waste characterization. The functional unit is related to the total amount of waste generated in 1 year expressed in metric tonnes. By the outcomes of the analysis, future management scenarios can be evaluated: recycling rate and cost estimations can be obtained.

### Scenarios’ analysis

#### Aim and scope

The aim of the scenarios’ analysis is to estimate the maximum and minimum recycling level that can be potentially achieved by a CDW management system. By these results, the average annual costs for a reference scenario are assessed. At the same time, indications about the optimal scenario can be provided. The following material flows have been considered: (i) the products obtained from the processing of recyclable materials; (ii) the amount of non-inert selected materials transferred to other facilities; (iii) and the amount of hazardous waste or rejects that should be disposed of. The economic analysis, in terms of operational and capital costs (OpEx and CapEx), were estimated for each scenario.

The system boundaries are related to the total amount of CDW potentially generated within the municipality of La Paz. The time scale refers to an average year, without any other specification. The system boundaries are focused on the treatment, recycling, secondary transportation (from the recycling facility to the final use), and final disposal. Treatment efficiencies and costs are related only to Latin American case studies found in the literature. Based on the literature, the typical products of a CDW are recycled aggregates of different size, which are suitable for various construction uses, generally of a non-structural type (Jadovski 2005; Sobral 2012). These products are obtained from recycling facilities that comprehend crushing, sorting, and sieving systems. These plants can be fixed (located in a specific site) or mobile (plants that are transported to generation sites), function of waste amounts, and characteristics. Non-structural building bricks can be also produced from recyclable aggregates. Therefore, the scenarios analysis considers the presence of fixed or mobile facilities, as well as source separated materials or mixed collection, with the production of blocks of recyclable aggregates or raw materials that can be used for road construction. The rejects or unrecyclable waste are considered to be disposed of in controlled sanitary landfills.

#### Scenario’s description

Three scenarios were considered, involving the implementation of a centralized block-making plant and the recovery of recyclable aggregates. The main differences are related to the source separation/collection and the treatment system:

Scenario 1 (S1): *Source separation and fixed recycling plants*. The first scenario involves the recycling of inert materials in fixed and centralized plants. Recyclable aggregates are obtained from source separation. After the selection of CDW at the source, recyclable aggregates are transported by the generators to a recycling facility, while hazardous waste and non-recyclable non-inert are transferred to a sanitary landfill. Medium-scale recycling plants, with the capacity of about 40,000–45,000 tonnes per year are introduced. The number of plants depends on the amount of waste estimated by the MFA. The outcome of the treatment plants foresees the production of four different recyclable materials. Big size aggregates are considered to be reused for road buildings while small size aggregates are sent to the centralized bricks-making plant. Rejects are deemed to be disposed of in a sanitary landfill, similar to the one available currently at municipal level. Finally, other recyclable materials source segregated are considered to be selected and recovered at the municipal recycling facility.

Scenario 2 (S2): *Source separation and mobile recycling plants*. Similar to S1, instead of fixed recycling facilities, this scenario also foresees the implementation of mobile treatment plants.

Scenario 3 (S3): *Mixed collection and fixed recycling plants*. The third scenario involves the implementation of fixed recycling facilities able to select mixed CDW. Therefore, more investments are considered to implement a centralized selection. These treatment plants should have a higher capacity than the ones implemented in S1 and S2. However, the number of the treatment plant is considered to remain the same, as well as the material recycling and final disposal.

On balance, S1 and S2 require a major effort from the CDW generators since the waste should be source segregated. On the other hand, the third scenario allows a raw collection from the owner of building activities, but more efforts should be spent at the treatment plant in order to select the waste to be recycled.

#### Inventory analysis

An investigation of the processes involved for both the crushing plants and the bricks production plants was carried out, through the case studies of CDW plants available in the scientific literature. According to the types of processes found within the bibliography, the specific products potentially obtained by the system were considered, both in sorting plants and in bricks production plants. By the analysis, it has estimated that about 95 ± 5% of the CDW inflow into the treatment plant and 98 ± 2% transported by the vehicles are effectively managed by the system, while the others are loose to the environment in the form of dust and sand. The products obtained in the fixed or mobile crushing plants are considered to be recycled.

The production capacity of a recycling plant is a parameter that depends on the type of plant, the technological level, and the selection methods, such as source segregation or mixed collection and sorting. The number of recycling facilities depends also on the scenario introduced by the analysis and the characteristics of the treatment plants. The production of bricks from recyclable aggregates correspond to a post-selection phase. For this reason, a single plant with a large production capacity is considered for each scenario. In other words, a fixed bricks production plant has been added to each scenario, in which the recycled aggregates are centralized. The characteristics and yield of products obtained according to the type of plant are reported within the supplementary materials for each scenario (Tables S3, S4, and S5).

### Economic assessment

A theoretical analysis of the economic costs involved in each scenario has been carried out. The following capital costs are considered: land acquisition, equipment and machinery acquisition, and indirect expenses of installation, assembly, and other engineering works. Therefore, each scenario is subdivided into two setups: considering the purchase of land and without its involvement.

At the same time, the following operational costs (OpEx) were assessed: salaries for human resources, social benefits, electricity consumption, fuel, water, input materials, and maintenance costs. Finally, the incomes obtained, thanks to the selling of secondary raw materials and products, are also involved within the analysis. The sum of the investments, operating costs, and incomes constitutes the total expenses that were uniformized in annual costs with a time horizon of 20 years.

Data has been collected from six Latin American case studies. Four plants from Brazil, one from México, and the one built in Bolivia were considered for collecting data about operational and capital costs. The plant built in Bolivia is the one with less capacity (about 19,000 tonnes per year) and recent construction (2021). The one with higher capacity is from México, with about 132,000 t y^−1^ (González 2016). Finally, the plants from Brazil have a capacity of 40,000 t y^−1^(Jadovski 2005; Sobral 2012), 30,750 t y^−1^ (Almeida and Chaves 2002), and 80,000 t y^−1^ (Jadovski 2005). Costs were uniformized in USD and reported to 2021.

Data conversion and actualization have been carried out through the housing development unit, a benchmark index that shows the daily evolution of prices that is calculated on the basis of the consumer price index, which has been published by the Bolivian national institute of statistics (INE). Similarly, process capacity has been also considered within the cost analysis, providing related proportion per tonnes of waste treated. Through these bibliographic sources, minimum and maximum values on investment costs and operating costs have been obtained. At the same time, the costs of land acquisition in different areas of the municipality of La Paz have been investigated through local documentation that refer to 2019.

#### CapEx

The estimated land cost in the municipality of La Paz is of about 274.9 ± 58.3 USD m^−2^. On average, it has been estimated that a fixed CDW recycling plant requires about 14.3 ± 3.2 USD t^−1^ y^−1^ as capital cost, while the indirect costs, which refer to installation costs, other facilities, project management and taxes, can be estimated to be about 4.17 ± 2.3 USD t^−1^ y^−1^. Similarly, a mobile trituration plant can cost about 5.75 ± 0.7 USD t^−1^ y^−1^ of capital expenditures and 1.73 ± 1.63 USD t^−1^ y^−1^ of indirect ones. Finally, the bricks production plant can cost 3.45 USD t^−1^ y^−1^ of capitals and 3.18 USD t^−1^ y^−1^ of variables. While the fixed and recycling plants refer to different plants in Latin America, the brick production plant is only related to the case study in La Paz (Bolivia).

#### OpEx

By the literature review, it has been estimated the average potential operational costs that should be afford for treating one tonne of CDW. For a CDW recycling facility (fixed or mobile) it has been estimated that about 4.03 E10^−4^ ± 1.12 E10^−4^ USD t^−1^ y^−1^ are required for human resources, with the addition of about 28% of salary costs. In addition, about 4.52 ± 1.45 kW t^−1^ of electricity, 9.42 ± 3.97 L t^−1^ of water, and approx. 3200 L^−1^ y^−1^ of fuel can be required. Considering the national costs of resources in 2020, equal to 0.1 USD kW^−1^ of electric energy, 1 USD m^−3^ of water, 0.53 USD L^−1^ of fuel, the net operational cost can be obtained for a recycling plant.

For a brick production plant, it can be estimated that about 4.27 E10^−4^ USD t^−1^_CDW_ are required for human resources (plus 28% of additional salary costs), while 2.35 kW t^−1^_CDW_ of electric energy, 3.20 USD t^−1^_CDW_ of fuel, and cement are needed. The cost of cement has been estimated to be about 1012 USD t^−1^ (Bolivian market). In addition, about 66% of the variable costs (energy, fuel, and water) should be added as maintenance costs.

Finally, transportation expenses have been considered within the analysis. In particular, the transportation of secondary raw materials, rejects, and other non-recyclable materials has been considered. About 3.2 ± 0.67 USD t^−1^ are required for the transportation of mixed CDW, 2.59 ± 0.54 USD t^−1^ are needed for transporting secondary raw materials, while transportation costs for rejects and non-recyclable materials have been considered equal to the average cost for the transportation of waste, equal to about 36.2 USD t^−1^. Finally, costs for final disposal have been estimated equal to the current cost for the disposal of municipal solid waste, which is about 14.5 USD t^−1^.

#### Sale of secondary raw materials and products

According to the Bolivian institute of statistics, the price of coarse aggregate is of about 17 USD m^−3^. As suggested by other authors, the price of recycled products should be less than 80% compared to a product made of virgin materials (Jadovski 2005). Therefore, for the current research, the price of recyclable aggregates has been estimated to be about 13.6 USD m^−3^. Considering a density for recycled aggregates of about 1025 t m^−3^ (Sobral 2012), and the change in the present currency rate to the USD, the final price of recycled coarse aggregate has been estimated to be equal to 13.18 USD t^−1^. Finally, in relation to the recycled bricks produced, the lowest price for a natural tile is of about 5.8 USD m^−2^ (Bolivian market). Considering the physical characteristics of the concrete block and the current exchange rate to USD, the price of the concrete block has been estimated to be equal to 26.67 USD t^−1^.

## Results

### Average constructed and demolished areas in La Paz

In general, timber is not a construction material in La Paz. Therefore, only concrete buildings have been involved within the analysis. Following the indications available from the local municipal government, on average, from 2013 to 2017, the private sector demolished about 139,756 ± 21,199 m^2^, without any specification in relation to the construction size, while construction of residential and non-residential areas are estimated to be about 757,237 ± 57,956 m^2^. Civil infrastructures built by the private sector are estimated to be about 25,571 ± 2296 m^2^. Regarding the public sector, about 28,594 ± 6471 m^2^ are estimated to be demolished, while the construction of residential and non-residential areas are estimated to be about 53,075 ± 13,119 m^2^. Finally, construction of civil infrastructures like paved areas, walls, water harvesting and management, and sewage systems, are estimated to be 54,725 ± 4563 m^2^, 70,647 ± 12,905 m^2^, 42,188 ± 9126 m^2^, and 11,213 ± 3232 m^2^ respectively. Data are reported in Table S6.

### Average WGR obtained from the scientific literature

#### Average construction WGR

**Fig. 2:**
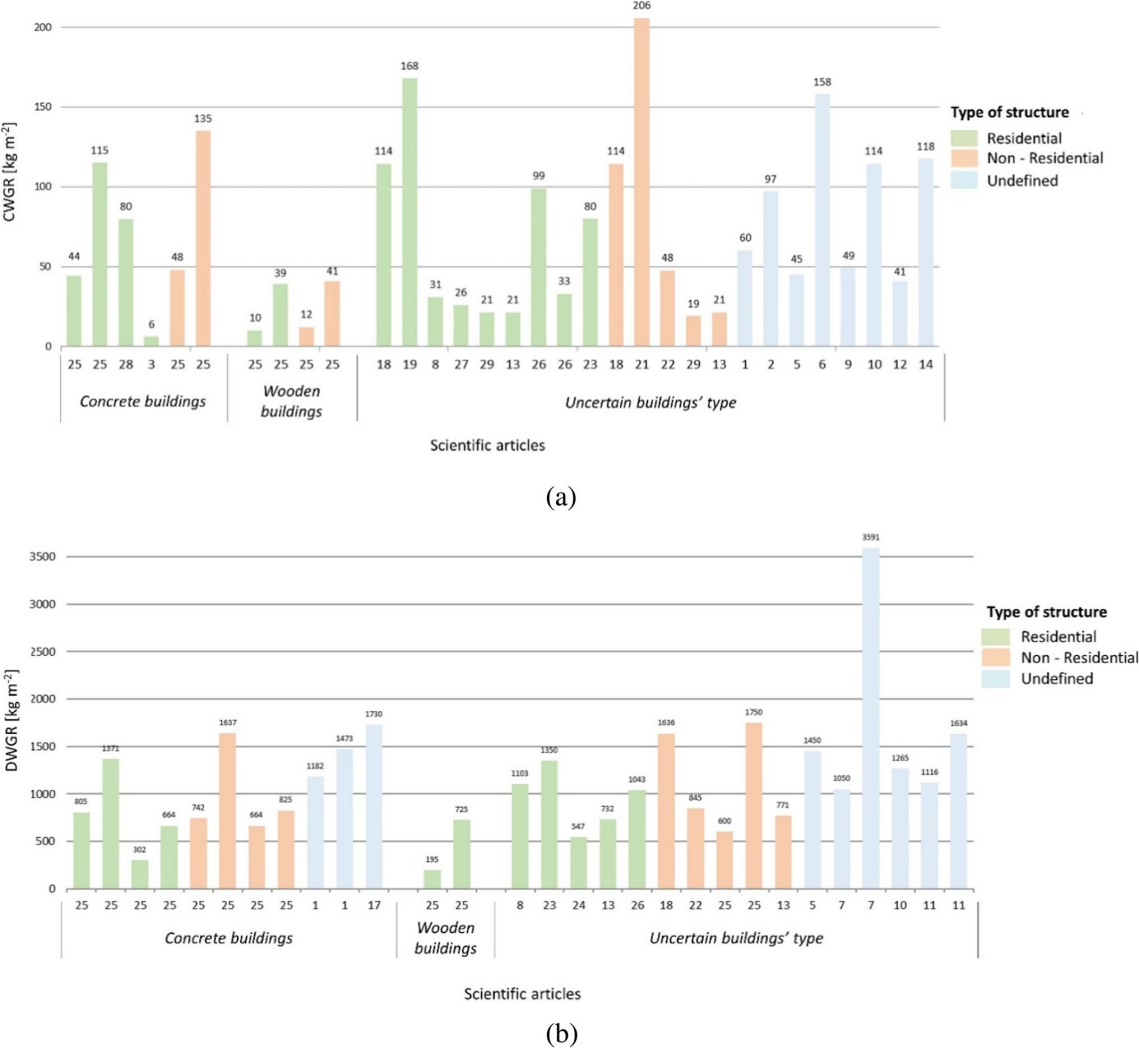
Results of the literature review: **a** construction waste generation rate (CWGR) and **b** demolition waste generation rate (DWGR)

Data collected from the scientific literature are reported in Fig. 2, grouped in function to the construction materials and size. In relation to CW (Fig. 2a), it is observed that the construction WGR (CWGR) of concrete structures with masonry present values always greater than 44 kg m^−2^, whereas the WGR values of wooden structures are less than 41 kg m^−2^. Therefore, data with unknown construction materials were grouped in relation to this information. By this group of values, it has obtained that residential buildings (made of concrete and masonry) have a CWGR of about 100.02 ± 14.65 kg m^−2^, non-residential 110.19 ± 29.65 kg m^−2^, and undefined buildings’ size an average of 99.30 ± 14.41 kg m^−2^.

The *t*-test has been implemented in order to evaluate if these average WGR are statistically different between constructions’ size. As expected, between timber and concrete structures, the statistical difference exists (t_[198]_ = 5.5, *p* < 0.005^***^), while this is not true among concrete structures with different size: there is not any statistical difference between non-residential and residential (t_[24]_ = 0.337, *p* = 0.743), unknown structure size vs. residential (t_[30]_ = 0.033, *p* = 0.974), and unknown structure size vs. non-residential (t_[20]_ = 0.337, *p* = 0.744). Thanks to this analysis, it can be stated that there is no need to differentiate WGR per structure size, and they can be unified into a single group in order to obtain a more reliable average value. An average CWGR of 102.6 ± 10.71 kg m^−2^ has been employed for the analysis.

#### Average demolition WGR

Similar considerations have been introduced for DW. Results are reported in Fig. 2b. High variability among values have been detected. In addition, very low information regarding demolition of wooden buildings are reported within the scientific literature. Only two values have been collected from wooden structures: 195 kg m^−2^ and 725 kg m^−2^, with an average of about 460 kg m^−2^. The average demolition WGR (DWGR) of wooden structures has been considered as referenced value for discriminating the building type. On the other hand, the value of 302 kg m^−2^ available from the concrete buildings is considered to be a low value in relation to the others in this group. However, the bibliography from which it was extracted (Mália et al., 2013) is reliable. For this reason, it continues to be considered. Based on these hypotheses, all WGR collected from uncertain buildings’ type have been considered within the concrete buildings’ group.

Thus, for concrete structures, the residential size has an average DWGR of 879.7 ± 121.2 kg m^−2^, non-residential 1052.3 ± 157.9 kg m^−2^, and the ones with undefined structure size 1362.5 ± 87.5 kg m^−2^. By the *t*-test, it has been found that statistical difference can be detected between the DWGR of residential structures, and the one where any detail is provided in relation to the size (t_[56]_ = 3.156, *p* = 0.006^**^). Differently, no statistical difference can be detected from non-residential vs. residential (t_[64]_ = 0.867, *p* = 0.399) and non-residential vs. unknown (t_[56]_ = 1.658, *p* = 0.118). Therefore, it could be deduced that data obtained from the undefined size of the structures cannot be involved within the analysis. Residential and non-residential size DWGR data were grouped, while the ones related to the unknown buildings’ not. On balance, the average DWGR used within the analysis is equal to 965.98 ± 98.8 kg m^−2^.

#### CDW generated from civil infrastructures

The WGR due to construction of civil infrastructures has been collected from de Magalhães et al. (2017). It should be highlighted that little is available within the scientific literature regarding waste generation from civil constructions: DW is not available. Regarding the typology of civil works constructions, the study highlighted the WGR for sewer systems, water harvesting, paved areas, fences, and temporary construction facilities. Original values were provided in cubic meters. Therefore, within the current research, data were converted in mass. By the analysis, it has been obtained that about 266.3 ± 29.9 kg m^−2^ of CW are generated for sewage systems, of which 96.9% recyclable aggregates, 2.2% other waste, and 0.9% hazardous waste; about 338.2 ± 36.4 kg m^−2^ of CW for fences, of which 99.6% recyclable aggregates, 0.3% others and 0.1% hazardous waste; about 244 ± 27.2 kg m^−2^ of CW for paved areas, with about 97.5% of recyclable aggregates, 1.8% others, and 0.7% hazardous materials; and finally, the sewage system counts for about 367.8 ± 41.7 kg m^−2^ of CW, with 96.3% aggregates, 2.63% others, and 1% hazardous.

#### Average CDW characterization

Data about CDW characterization has been reported in three groups: generated from DW, from CW, and the ones obtained from mixed CDW. Data related to characterization has been uniformized in terms of %wt. Therefore, percentage in volumes were converted in order to obtain representative and comparable values. Results of CDW characterization data collection are reported in supplementary materials (Table S7). Waste characterization has been grouped in terms of recyclable aggregates, wood, metals, gypsum, and others.

The generation of recyclable aggregates in the construction activity of buildings with a concrete structure is of about 76.3 ± 9.8%, while the demolition activity generates about 61.5 ± 12.6%. This represents the waste fraction more available within both CW and DW. By the statistical analysis, it has been found that there is not any difference among CW and DW composition (t_[8]_ = 0.813, *p* = 0.447). However, comparing DW with mixed CDW composition, a statistical difference has been found (t_[20]_ = 2.325, *p* = 0.045^*^). Therefore, for avoiding data unreliability, the specific characterization of CW and DW has been considered different and it has been maintained separated, while waste characterization of mixed CDW was not employed.

This hypothesis has been considered valid also for the other materials since they represent the residues less available. The generation of wood materials in the construction activity is of about 6.8 ± 3.6%, while in the demolition activity 10.7 ± 8.5%. Metals are estimated to be generated for about 3.9 ± 2.1% for construction activities, and 15.9 ± 11.6% for demolition. Finally, gypsum is considered to be about 4.3 ± 3.3% for construction, and 0.4 ± 0.4% for demolition, while others are estimated to be 4.4 ± 2.8% for construction and 11.4 ± 4.4% for demolition. These results are used within the MFA.

#### Correlation analysis

**Fig. 3:**
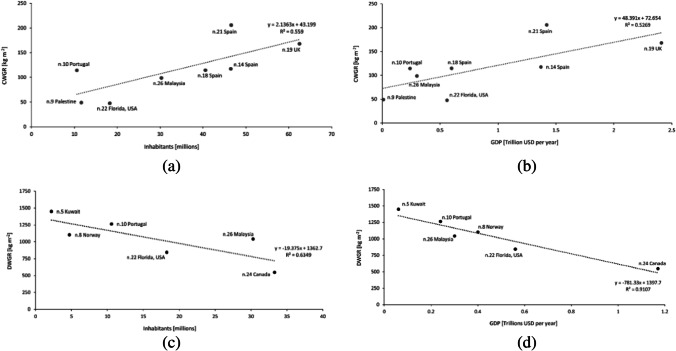
Linear regression of the WGR in relation to the number of inhabitants and the national GDP: **a** Construction waste generation rate (CWGR) per GDP, **b** CWGR per inhabitants, **c** Demolition waste generation rate (DWGR) per GDP, and **d** DWGR per inhabitants

Pearson correlation statistic has been implemented in order to obtain additional data in relation to the variability of the WGR. In particular, CWGR and DWGR were correlated to the national or regional inhabitants and the GDP. The reason behind the analysis is that a correlation might exist between GDP, number of inhabitants, and WGR, similar to municipal solid waste (Grazhdani 2016; Zia et al. 2017). A total of eight studies were collected about the CWGR, while six studies were related to national average DWGR. Results are presented in Fig. 3.

A correlation has been found for all the groups. In particular, a significant positive correlation has been found in relation to the CWGR both for the number of inhabitants (*r* = 0.75, *p* = 0.033^*^) and the GDP (*r* = 0.73, *p* = 0.042^*^). Therefore, the initial hypothesis has been confirmed. The higher the GDP and number of national inhabitants, the greater the CWGR. Results are presented in Fig. 3a and b, where a linear regression is also given. Again, a correlation has been detected also in relation to the DWGR. However, in this case, a negative correlation has been found for the GDP (*r* =  − 0.954, *p* = 0.003^**^), while for the number of inhabitants, a negative correlation has been found, but with a weak significance (*r* =  − 0.8, *p* = 0.057). The linear regression is reported in Fig. 3c and d. It means that, the higher the income level, the lower the generation of waste due to demolition activities.

This statistical analysis can be used in order to evaluate the reliability of the data used for the MFA. Considering that Bolivia, currently, has a GDP equal to about 0.04 trillion of USD and a number of inhabitants of about 11 million, in function of the linear regression presented, it can be stated that the average potential national CWGR can be around 66.7 to 74.6 kg m^−2^, while the DWGR can be around 1366 kg m^−2^. The hypothesis can be that, for Bolivia, these data are mainly representative for the urban areas like La Paz, while they are not for the rural ones. Therefore, comparing the average values employed for the MFA, data related to the CWGR used within the research (about 102 ± 10 kg m^−2^) are quite similar to the one expected at national level by the regression statistics while the DWGR are lower (about 965 ± 10 kg m^−2^) than the ones that can be obtained by the regression analysis. On balance, the average WGR used within the research can underestimate the overall amount of waste that can be generated by the system; therefore, the potential amounts of CDW produced at municipal level can be even bigger.

### Waste flow analysis of La Paz

**Fig. 4:**
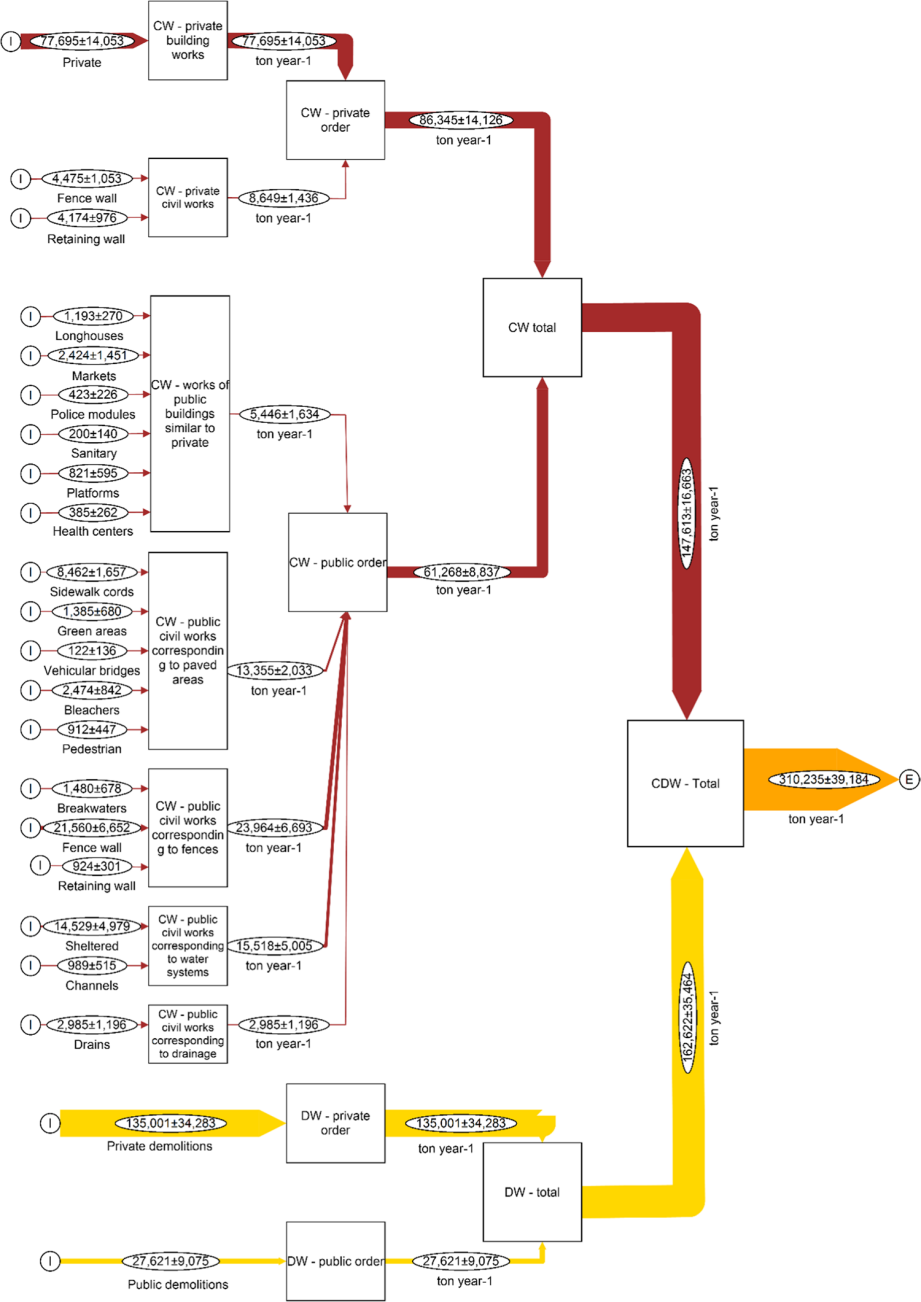
Waste flow analysis. Construction waste (CW) and demolition waste (DW) amounts generated in La Paz. CDW: construction and demolition waste

The general MFA related to the CDW potentially generated in La Paz is reported in Fig. 4. According to the results, the municipality of La Paz can potentially generate approximately 310,234 ± 39,184 t_CDW_ y^−1^. On the other hand, the current CDW amount managed by the municipality of La Paz is approximately 127,511 ± 8,165 m^3^ of CW and 121,380 ± 17,838 m^3^ of DW, making a total of 248,892 ± 19,618 m^3^ of CDW generated per year. With an average density of 830.6 kg m^−3^ and giving it a variability of 20%, the estimated result can be about 206,730 ± 44,441 t_CDW_ y^−1^. It means that, compared with the results obtained by the present study, the CDW amount estimated by the local municipality are 67 ± 17% of the one estimated by the MFA. Therefore, local municipality is potentially underestimating the amount of CDW generated in La Paz.

Following the indications provided by the MFA, the private order potentially generates about 221,346 ± 37,080 t_CDW_ y^−1^. On the other hand, the public potentially generates about 88,889 ± 12,667 t_CDW_ y^−1^. This information reveals that the private order generates more CDW than the public order, around 71.4 ± 15%. In relation to the total amount of CDW, the contribution related to CW and DW are similar: around 47.6 ± 8.1% and 52.4 ± 13.2% for CW and DW, respectively.

Finally, the characterization analysis (Fig. S1) allows identifying that about 229,235 ± 30,765 t y^−1^ of recyclable aggregates are generated, which is 73.9 ± 13.62% of the total, and non-inert recyclable can be estimated to be around 80,692 ± 22,991 t y^−1^, corresponding to 26 ± 8.1%, while hazardous waste represent a minimal part, with about 306.7 ± 59.2 t y^−1^, corresponding to 0.1 ± 0.02%.

### Scenarios’ analysis

**Fig. 5:**
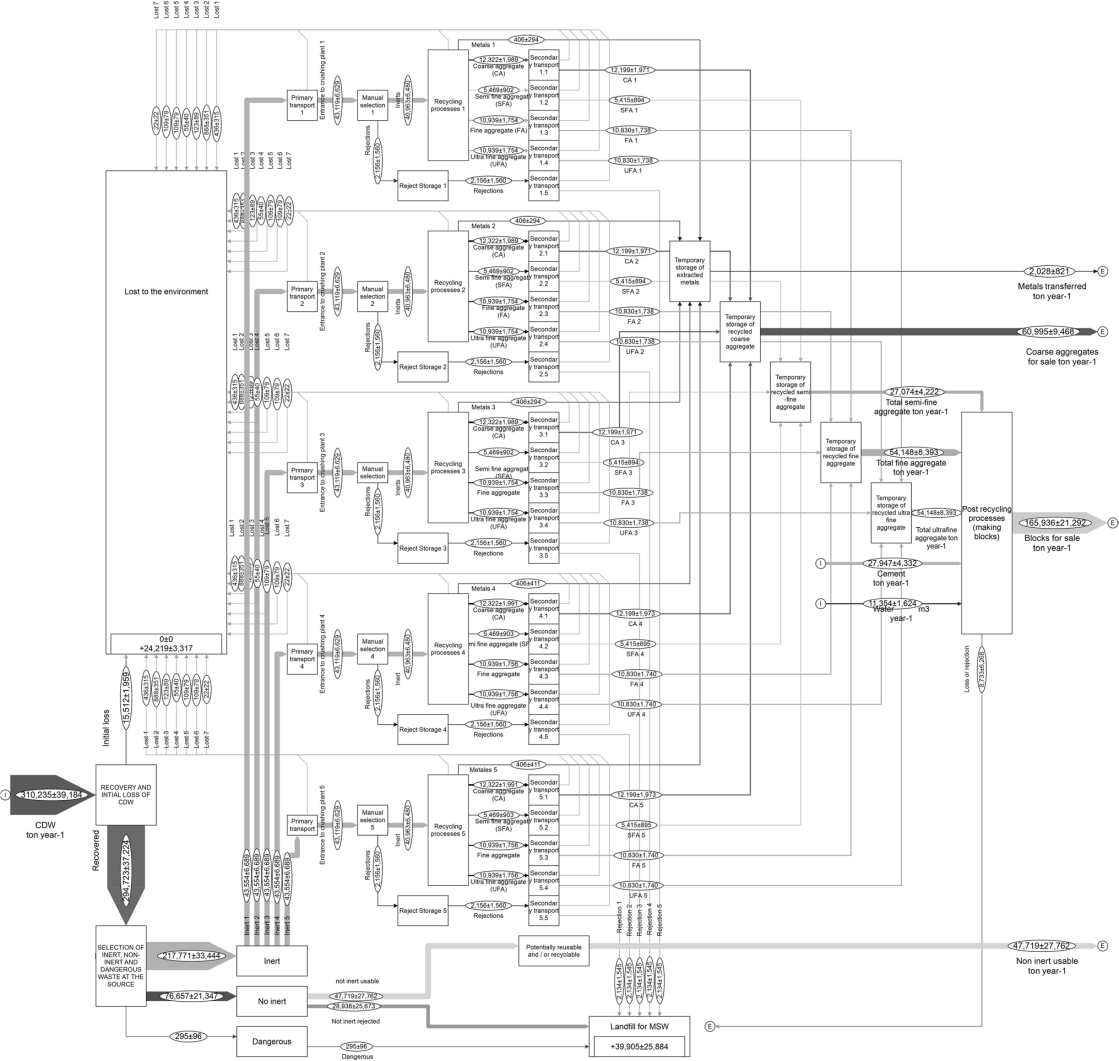
Waste flow analysis of Scenario 1

The CDW MFA have been implemented for each scenario. The result of the MFA related to S1 is reported in Fig. 5 (Figs. S2 and S3, related to S2 and S3, are reported in supplementary materials). In the first scenario, the inflow for each fixed treatment plant is equal to 43,119 ± 6,629 t y^−1^. Totally, the waste inflow into the plants is equal to about 215,593 ± 33,145 t y^−1^. The annual amount of coarse aggregated potentially produced is of about 60,995 ± 9,468 t y^−1^, while about 165,936 ± 21,292 t y^−1^ of concrete blocks are made, equivalent to 5,028,366 ± 645,220 block units (from 4.38 to 5.67 million blocks per year). On balance, S1 could allow producing coarse aggregates corresponding to 26.9 ± 5% of the total recyclable aggregates generated, useful as sub-base of soil in pavement construction, and a production of concrete blocks corresponding to 73.1 ± 12% that can be used for tiling floors that would cover around 1,257,084 ± 161,305 m^2^ y^−1^.

In S2, the annual CDW processing capacity of each fixed sorting plant is equal to 41,065 ± 6,313 t y^−1^, while for a mobile crushing plant it is 31,110 ± 4,778 t y^−1^. Totally, about 216,526 ± 23,752 t y^−1^ of CDW can be treated per year. The annual amount of aggregates generates is equal to 97,970 ± 15,188 t y^−1^, and about 122,107 ± 15,733 t y^−1^ of concrete blocks are produced, equal to 3,700,216 ± 476,760 block units per year. Therefore, S2 allows producing about 44.5 ± 8.0% of recyclable aggregates for pavement construction and about 55.5 ± 8.9% of recycled blocks, useful for tiling floors that would cover about 925,054 ± 119,190 m^2^ y^−1^.

Finally, the annual CDW processing capacity of each fixed crushing plant for S3 is equal to 58,297 ± 7,375 t y^−1^. In total, the 5 fixed plants allows treating about 291,484 ± 36,874 t y^−1^. Due to the mixed collection system, the amount of recyclable aggregates generated is estimated to be lower compared to S1 and S2. About 59,861 ± 8,334 t y^−1^ of coarse aggregate, and 162,852 ± 18,915 t y^−1^ of concrete blocks are produced, which counts about 4,934,923 ± 573,171 block units (from 4.36 to 5.51 million blocks per year). The production of coarse aggregates correspond to 26.9 ± 4.6%, and 73.12 ± 11.13% of concrete blocks covering 1,233,731 ± 143,293 m^2^ y^−1^.

### Comparison of scenario’s performances

**Fig. 6:**
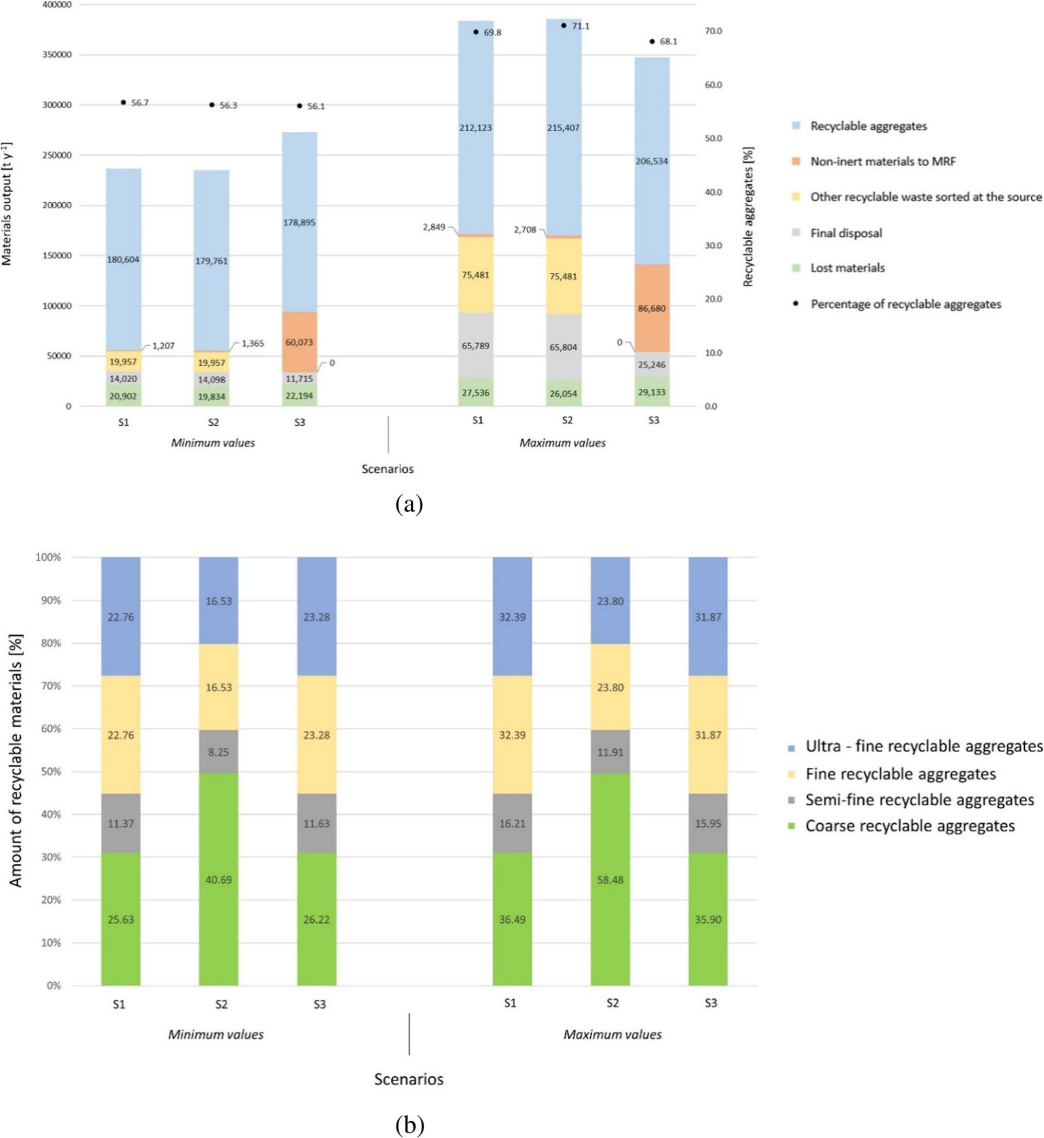
Scenarios’ comparison in relation to **a** the material flow and recycling rate and **b** the fraction of recyclable materials generated by the system

In Fig. 6a, the total amount of waste treated, and the percentage of waste recycled are reported. The three proposed scenarios have similar annual amounts of waste lost to the environment, function of the transportation, and the open MRF. The waste rejected by the system is greater in S1 and S2 compared to S3, although it depends on the waste flow transported to the MRF. The annual amount of recycled aggregates produced is very similar in the three scenarios. The production of recycled aggregates with respect to the total inflow is of about 63.3% for S1, 63.7% for S2, and 62.1% for S3. These findings reveal that the last scenario is the one with the lowest production of recyclable aggregates, although with a very narrow difference between the other scenarios. Therefore, the recycling rate cannot be the reference parameter for choosing the most affordable scenarios.

On the other hand, there is great variability of minimum and maximum values for S1 and S2 compared to S3, due to the dependence on the quality of the source segregation. The amount of recyclable aggregate production with respect to the total CDW generated is from 56.8 to 69.9% for S1, from 56.3 to 71.1% for S2, and from 56.1 to 68.2% for S3. This last scenario being the one with the lowest production, although with a lower difference from lower and higher values compared to the other scenarios.

In Fig. 6b, the specific amount (in percentage) of recyclable aggregates generated by the system are reported. There is a noticeable difference in the production of recycled aggregates in S2. It is observed that coarse aggregates are mainly produced in this scenario, with a range of 40.7–58.5%, unlike S1 and S3 that generate about 25.6–36.5% and 26.2–35.9%, respectively. Thus, reducing for S2 the average production of semi-fine aggregates to 8.25–11.9% and of fine and ultra-fine aggregates to 16.5–23.8% each. This is due to the use of on-site mobile crushing plants, which produce only coarse aggregates.

### Costs analysis

**Fig. 7:**
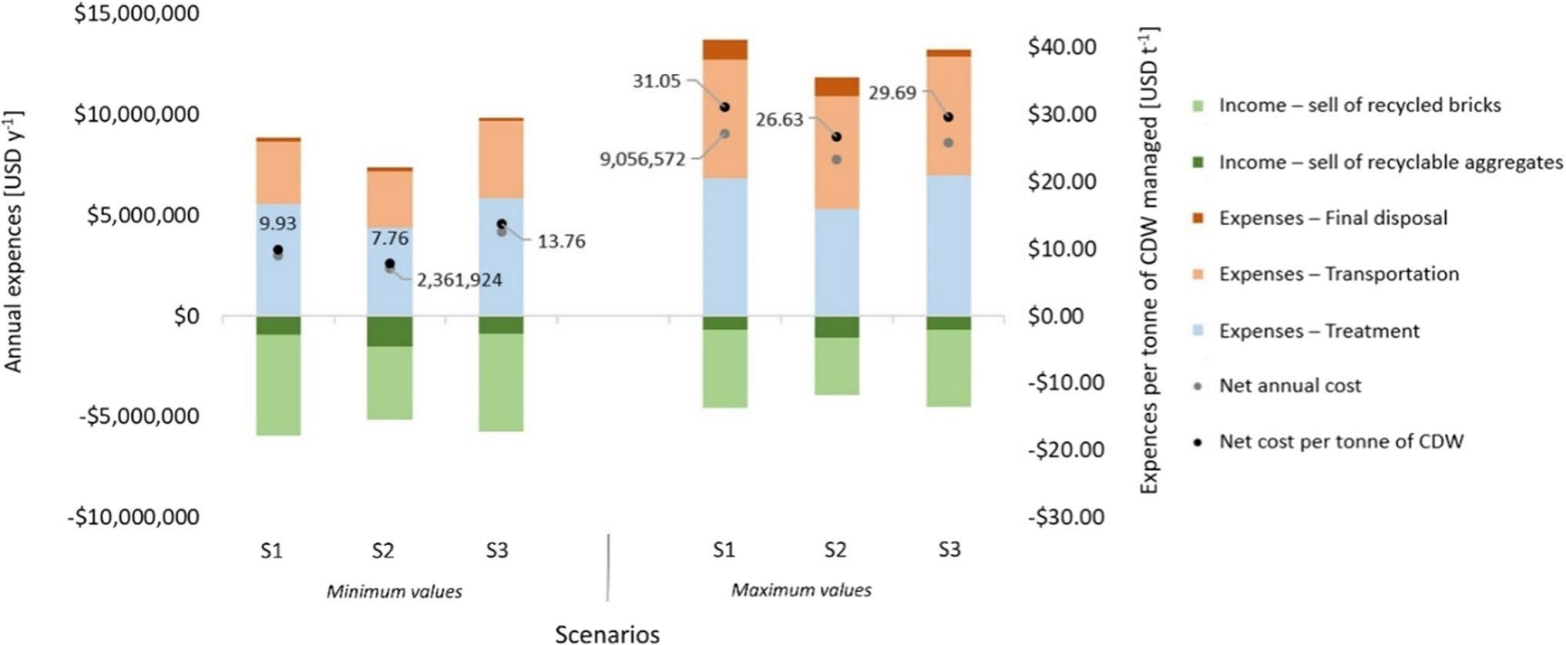
Results of the costs analysis involving land acquisition

In Fig. 7, the result related to the cost analysis is reported. It highlights the annual expenses that the municipality of La Paz should potentially afford considering the investments related to land acquisition. By the analysis, it has estimated that about 2.3 to 9 million dollars can be required for CDW management, which is equal to about 7.8 to 31.1 USD per tonne. The maximum expenses can be due to S1, where fixed recycling facilities should be introduced, with a maximum cost of about 9 million dollars per year, equal to about 31.1 USD per tonne of waste managed. It can be stated that, in the best case, selective collection at the source allows reducing management costs, due to the higher income for waste recycling. However, in the worst case, selective collection with fixed sorting plants cannot be considered the best option. S2 is the most reasonable scenarios in terms of cost affordability. The optimum can be therefore obtained with the hybrid implementation of on-site and off-site sorting systems, allowing the reduction of the costs, and anyhow providing effective results in terms of recycling rates.

On balance, in general, the best scenario is obtained when higher source sorting efficiency is achieved, reaching 86% of selective collection of recyclable aggregates. Similarly, in the best case, about 56.3% of the recyclable aggregates are used for recycling. At the same time, absence of CDW lost to the environment should be obtained, as well as the maximum amount of metals selection and recycling, equal to about 9.3%. In addition, in the best case, the maintenance cost is reduced of about 13% compared to the worst case, and the investment decreased of about 19%. Finally, in the best case, the income due to recyclable waste achieved 23% more compared to the worst case. This variability depends on the market, the price of virgin materials, and the economic development of the country, among others. The graphs related to the specific costs per scenario are reported within the supplementary materials (Fig. S4).

By these results, the average fee that the generator should pay for CDW management can be estimated. In particular, considering the average construction and demolition WGR, and the average net cost per tonne of waste treated, the average fee per square meter can be found. By the study, it can be defined that about 7.4–36.6 USD m^−2^ are required for managing CDW, which is about 0.6–4.3% of the current cost of an average flat in the center of La Paz (about 860–1300 USD m^−2^). Depending on the scenario, with the hypothesis that the expenses should be < 1% of the current costs, they can be considered affordable.

## Discussion

The research demonstrates that the CDW management system requires high investments and operational costs. However, it can also be considered a great source of income for private companies and recycling facilities: other studies report that a recycling company can earn almost USD 250,000 in a year by recycling CDW (Oliveira Neto and Correia 2019).

MFA is an approach widely used in the scientific literature for quantifying CDW in cities. For example, in India, it has been demonstrated that the CDW generation was between 112 and 431 million tonnes in 2016, depending upon the assumptions, which are orders of magnitude higher than official records (Jain et al. 2021). Similar results has been found in the current research carried out in La Paz, highlighting that the current system is probably underestimating the amount generated at municipal level.

### Challenges in managing CDW in Bolivia

Validation of the results obtained by the analysis is very difficult in La Paz. Local data are not always reliable. Therefore, waste flow analysis (although implemented by secondary data) can support analysis of local requirements. In addition, pilot projects can highlight the main barriers for starting a circular model, also involving the informal recycling sector active and widespread in the city (Ferronato et al. 2020a). Investments required for treating CDW are very high for a low-income economy. La Paz might suffer its implementation due to the lack of financial sustainability. However, it was important to estimate them in order to give the order of magnitude to decision makers. The main problem is: “how can we move towards a circular CDW management if financial resources are lacking?” The research demonstrates that on-site waste treatment and sorting should be prioritized. However, “how can we provide incentives to local generators?” These are questions open to future research and assessment.

### Policy implications and recommendations

The research reported in this paper highlights the importance of data collection and assessment in CDW management. The first step that decision makers should consider is to start by small-scale treatment plants that can provide the order of magnitude of local costs and the market of secondary raw materials. Appropriate disposal should be incentivized in order to have ideas about the amount of waste generated at municipal level, giving more indications about the effective policies and investments required for improving CDW management systems.

The market of recyclable aggregates should be explored, and stakeholders potentially interested in acquiring the raw material should be found. The development of new technologies should be regulated with appropriate norms and laws. The inclusion of CDW generators is recommended since they are the ones that can benefit of the system, or that can be affected by an erroneous CDW management. New taxes should be planned to be charged to CDW generators that turns into a higher cost of buildings: although such a cost has been demonstrated to be potentially negligible compared to the current one. Capacity building and know how should be also developed by the time, in terms of taxes organization and collection, regulation and management, technological development, and operational issues.

Monitoring and assessment of CDW characteristics and amount should be guaranteed, organizing laboratories that are expert in the field of construction and demolition, in order to give chances to the producers to certify the aggregates generated by the system. Other authors similarly suggest to understanding the dynamics and mobility during the life cycle of CDW, establishing CDW disposal charging system, and to developing advanced methods to assess the CDW management performance (Wu et al. 2019). The research introduced in this paper is the first step in La Paz for better planning future policies of CDW management and recycling.

### Limits of the analysis and future research directions

The research conducted in La Paz mainly employed secondary data. Statistical analyses were performed for better estimating the reliability of the average results obtained and range of values were used for estimating the costs and the potential recycling rates. Sample size is limited due to the low amount of scientific articles published in the last decades about WGR and CDW characterization. Therefore, it represent a limit and a future direction of research to provide more reliable and effective information related to CDW generation and management. The current research provided evidence about this requirement.

The linear regression from 6 to 8 samples was conducted to evaluate potential correlations and compare the WGR obtained by the waste flow analysis. The correlation based on GDP and inhabitants can be reasonable for reaching an estimate of the WGR to be used in developing cities. However, it cannot be strong enough to guarantee the existence of a strong correlation. Therefore, it is recommended to collect more data related to WGR and other variables, in order to better assess and confirm the potential correlation found in this research: the analysis provided the first results in the scientific literature regarding this potential linkage.

Finally, studies that analyzed the factors affecting the WGR should be better evaluated and included. This involve the awareness of construction personnel in the region to waste management issues, the design, and technologies used, the quality of workmanship, construction methods used, supervision, among others. Reviewing this literature would better help estimating the WGR of Bolivia since the statistical analysis performed and related on a limited sample might lead to faulty conclusions. This can be a future direction of research in order to confirm the results and the hypothesis introduced in this scientific research.

## Conclusions

The research performed in La Paz provides the first attempt to estimate the CDW flows and costs to decision makers and SWM practitioners. The study provided several findings that allows delivering likewise indications and suggestions of improvements.

Potentially, the municipality generates two times more CDW than the amounts that are currently estimated by the municipal government. It represents the starting point for considering the introduction of CDW collection systems able to gather and register the amount of CDW generated at municipal level: the idea is that “you cannot plan what you cannot measure.”

Second, the review reported in this research, first step of the analysis, underlined that there is a potential correlation between GDP, number of inhabitants, and WGR. This is good news and novel contribution to the scientific literature since it provides a first indication to quantify the CDW potentially generated at international level, especially in developing countries.

Third, the research introduces the most convenient scenario at an economic level, which is a hybrid treatment system (mobile and centralized) that considers the treatment in the place of generation with a source segregation. The main issue is: “is it possible in the context of La Paz?” Geographic information systems and spatial analysis can support such a recommendation.

In general, the cost of the system is estimated between about 7.8 to 31.1 USD per tonne of CDW managed, for a total annual cost of 2.3 to 9 million dollars per year (in 20 years-time horizon) depending on the management scenario. With the best scenario, a fee less than 1% of the current cost per square meter of an average flat of La Paz can be achieved: it can be considered affordable for the local economy.

In conclusion, the research demonstrated that the costs of CDW are very high, but that an appropriate management is possible. Construction and demolition waste should be prioritized in developing countries in order to recover materials and reducing waste open dumping. Pilot projects can be implemented as first step, with the aid of the cooperation with national and international actors, combining on-site and off-site treatment plants. The results of this research are the starting point of a circular economy of CDW in La Paz, a contribution to support sustainable development in developing countries at an international level.

## Supplementary Information

Below is the link to the electronic supplementary material.

Supplementary file1 (DOCX 23 KB)

Supplementary file2 (DOCX 15 KB)

Supplementary file3 (DOCX 21 KB)

Supplementary file4 (DOCX 16 KB)

Supplementary file5 (DOCX 18 KB)

Supplementary file6 (DOCX 19 KB)

Supplementary file7 (DOCX 24 KB)

Supplementary file8 (PNG 1173 KB)

Supplementary file9 (JPG 3085 KB)

Supplementary file10 (JPG 4424 KB)

Supplementary file11 (JPG 280 KB)

## Data Availability

The datasets generated during and/or analyzed during the current study are available from the corresponding author on reasonable request.

## Abbreviations

CDW: Construction and demolition waste
CW: Construction waste
DW: Demolition waste
MRF: Material recycling facilities
MFA: Material flow analysis
WGR: Waste generation rate
CWGR: Construction waste generation rate
DWGR: Demolition waste generation rate
SWM: Solid waste management
INE: National Institute of Statistics
GDP: Gross domestic product
