# Supplementary material for: Construction and demolition waste recycling in developing cities: management and cost analysis
Source: Environ Sci Pollut Res Int. 2022 Nov 7;30(9):24377–97. doi: 10.1007/s11356-022-23502-x (PMC9938826; doi:10.1007/s11356-022-23502-x)
Supplement: Supplementary file 1 — Supplementary file1 (DOCX 23 KB) [file 11356_2022_23502_MOESM1_ESM.docx]

**Table S1**: Waste generation rate (WGR) collected from the scientific literature. N.A.: not available/undefined; CWGR: construction WGR; DWGR: Demolition WGR.

| **N.** | **Reference** | **City, country, or region** | **Construction type** | | **CWGR** | **DWGR** |
| --- | --- | --- | --- | --- | --- | --- |
|  |  |  | **Structure** | **Dimension** |  |  |
| *1* | Ram y Kalidindi (2017) | Chennai, India | Concrete | N.A. | - | 1182 |
|  |  |  |  |  | - | 1473 |
|  |  |  | N.A. | N.A. | 60 | - |
| *2* | Paz y Lafayette (2016) | Brazil | N.A. | N.A. | 97 | - |
| *3* | Lu et al. (2011) | Shenzhen, China | Concrete | Residential | 6.038 | - |
| *4* | Poon et al. (2004b) | Hong Kong, China | N.A. | N.A. | 0.14* | - |
|  |  |  |  |  | 0.20* |  |
| *5* | Kartam et al. (2004) | Kuwait | N.A. | N.A. | 45 | 1450 |
| *6* | De Melo et al. (2011) | Lisboa, Portugal | N.A. | N.A. | 158 | - |
| *7* | Bernardo et al. (2016) | Lisboa, Portugal | N.A. | N.A. | N.A. | 1050 |
|  |  |  |  |  | N.A. | 3591 |
| *8* | Bergsdal et al. (2007) | Norway | N.A. | Residential | 30.77 | 1103.25 |
| *9* | Al-Sari et al. (2012) | Palestina | N.A. | N.A. | 49 | - |
| *10* | Coelho y de Brito (2011b) | Portugal | N.A. | N.A. | 114.3 | 1265 |
| *11* | Ding y Xiao (2014) | Shanghai, China | N.A. | N.A. | - | 1116 |
|  |  |  |  |  | - | 1634 |
| *12* | Li, J. et al. (2013) | Shenzhen, China | N.A. | N.A. | 40.7 | - |
| *13* | [EPA (2009)](https://www.epa.gov/sites/production/files/2017-09/documents/estimating2003buildingrelatedcanddmaterialsamounts.pdf) | USA | N.A. | Residential | 21.43 | 732.24 |
|  |  |  |  | Non-residential | 21.19 | 771.29 |
| *14* | Sáez et al. (2014) | España | N.A. | N.A. | 117.5 | - |
| *15* | Solís et al. (2009) | España | N.A. | N.A. | 0.31* | - |
|  |  |  |  |  | 1.27* | - |
| *16* | Llatas (2011) | España | N.A. | N.A. | 0.14 | - |
| *17* | Srour et al. (2013) | Beirut, Lebanon | Concrete | N.A. | - | 1730 |
| *18* | Mañá et al. (2000) | España | N.A. | Residential | 114.47 | - |
|  |  |  |  | Non-residential | 114.47 | 1635.97 |
| *19* | SMARTWaste (2010) | UK | N.A. | Residential | 168.05 | - |
| *20* | Myhre (2000) | Norway | N.A. | Non-residential | -** | - |
| *21* | Ortiz et al. (2010) | España | N.A. | Non-residential | 205.89 | - |
| *22* | Cochran et al. (2007) | Florida, USA | N.A. | Non-residential | 47.6 | 845 |
| *23* | Lage et al. (2010) | Galicia, España | N.A. | Residential | 80 | 1350 |
| *24* | Metro (2008) | Canada | N.A. | Residential | N.A. | 547 |
| *25* | Mália et al. (2013) | European Union | Concrete | Residential | 44 | 805 |
|  |  |  |  |  |  | 1371 |
|  |  |  |  |  | 115 | 302 |
|  |  |  |  |  |  | 664 |
|  |  |  |  | Non-residential | 48 | 742 |
|  |  |  |  |  |  | 1637 |
|  |  |  |  |  | 135 | 664 |
|  |  |  |  |  |  | 825 |
|  |  |  | Timber | Residential | 10 | 195 |
|  |  |  |  |  | 39 | 725 |
|  |  |  |  | Non-residential | 12 | - |
|  |  |  |  |  | 41 | - |
|  |  |  | N.A. | Non-residential | - | 600 |
|  |  |  |  |  | - | 1750 |
| *26* | Mah et al. (2016) | Malaysia | N.A. | Residential | 98.8 | 1042.82 |
|  |  |  |  |  | 32.86 |  |
| *27* | Umar et al. (2018) | Malaysia | N.A. | Residential | 25.79 | - |
| *28* | Mercader y Ramírez (2013) | Sevilla, España | Concrete | Residential | 79.79 | - |
| *29* | Kofoworola y Gheewala (2009) | Thailand | N.A. | Residential | 21.38 | - |
|  |  |  | N.A. | Non-residential | 18.99 | - |

*(*) units in m^3^ m^-2^*

*(**) value obtained from additional sources*
