# Supplementary material for: Construction and demolition waste recycling in developing cities: management and cost analysis
Source: Environ Sci Pollut Res Int. 2022 Nov 7;30(9):24377–97. doi: 10.1007/s11356-022-23502-x (PMC9938826; doi:10.1007/s11356-022-23502-x)
Supplement: Supplementary file 2 — Supplementary file2 (DOCX 15 KB) [file 11356_2022_23502_MOESM2_ESM.docx]

**Table S2**: Densities of construction and demolition waste (CDW) materials

| n. | Region, city, or country | Densities [kg m^-3^] | | | | | | | | | | | | | |
| --- | --- | --- | --- | --- | --- | --- | --- | --- | --- | --- | --- | --- | --- | --- | --- |
|  |  | Concrete | Masonry | Bricks | Bricks and masonry | Sand | Gypsum | Ceramics | Timber | Metals | Plastics | Glass | Paper and  cardboard | Hazardous | Mixed CDW |
| ***1*** | Chennai, India | 2300 |  |  | 1920 |  |  |  |  |  |  |  |  |  |  |
| ***17*** | Beirut, Lebanon | 1670 |  |  |  |  |  |  |  |  |  |  |  |  |  |
| ***25*** | General |  |  |  |  |  | 207.7 |  | 178 | 900 | 13 | 2500 |  |  | 830.6 |
| ***26*** | Malaysia | 1260 |  | 1400 |  | 1560 | 330 | 1170 | 390 | 900 |  |  |  |  |  |
| ***18*** | Spain |  |  |  |  |  |  |  |  |  |  |  |  | 600 |  |
