# Supplementary material for: Construction and demolition waste recycling in developing cities: management and cost analysis
Source: Environ Sci Pollut Res Int. 2022 Nov 7;30(9):24377–97. doi: 10.1007/s11356-022-23502-x (PMC9938826; doi:10.1007/s11356-022-23502-x)
Supplement: Supplementary file 3 — Supplementary file3 (DOCX 21 KB) [file 11356_2022_23502_MOESM3_ESM.docx]

**Table S3:** Inventory of Scenario 1 (S1)

| **SITE OR PROCESSING STAGE** | **OPERATIONAL STAGE** | **PROCESSES INVOLVED** | **MACHINERY AND / OR EQUIPMENT** | **DETAILS** | **PERFORMANCE** |
| --- | --- | --- | --- | --- | --- |
| Generating source | Separation and collection | Recovery and initial loss of generated RCDs | - | Recovery of RCD generated of 95.00%. Considering an inevitable loss to the environment of 5%. | 95.00 ±0.00% |
|  |  | Manual selection of inert, non-inert and hazardous waste at the source of generation | - | Selection of inert, non-inert and hazardous waste generating sources, corresponding to an approximate municipal level of 73.89 ± 12.19%, 26.01 ± 7.61% and 0.10 ± 0.03% respectively, of the total generated. | 73.89 ±12.19% |
|  |  | Manual selection of non-inert waste that can be used and rejected at the source | - | Selection at the source of non-inert waste that can be used on the site (metal and wood) and rejected (plaster, paper, glass, others), corresponding to an approximate at the municipal level of 62.25 ± 62.25% and 37 , 75 ± 35.64%, respectively, of the total of non-inert generated. | 62.25 ±62.25% |
| Fixed crushing plant | Transport | Primary transport of selected inert | Front loader and dump trucks with open hopper | Transport of the selected total inert waste to the 5 crushing plants. Whose amount is distributed equitably at 20% for each plant. Of the quantities already transported, it is considering a loss to the environment due to loading, unloading and / or journey, of 1.00 ± 1.00%. | 99.00 ±1.00% |
|  |  | Transport of rejected non-inert and hazardous waste | Front loader and dump trucks with open hopper | Transportation of rejected non-inert waste and hazardous waste to a municipal sanitary landfill, without considering a loss to the environment due to loading, unloading or journey. | 100.00 ±0.00% |
|  | Recycling | Collection of inert transported and received in plants | Vibrating pyramidal mailbox | Inert reception that will be fed in the following processes. It is not considered lost. | 100.00 ±0.00% |
|  |  | Manual selection of recyclable inert and rejected inert | Low speed vibrating feeder | Selection of inert received, manual extraction of impurities considered as rejection corresponding to 5.00 ± 5.00% of the total of inert entered, approximately. | 95.00 ±5.00% |
|  |  | Pre-crushing | Pneumatic hammer | Reduction in the size of inert admitted greater than 30 cm in average diameter if they exist. Considering a loss to the environment due to the generation of dust, of 0.5 ± 0.5% of the total pre-crushed. | 99.50 ±0.50% |
|  |  | Primary crushing | Jaw crusher | Crushing of inert smaller than 30 cm to sizes approximately 20 mm in average diameter. Considering a loss to the environment due to the generation of dust, of 0.5 ± 0.5% of the total primary crushing. | 99.50 ±0.50% |
|  |  | Ferrous Metal Separation | Electromagnet | extraction of steel chips from reinforced concrete waste. Considering an extraction of 1.00 ± 1.00% of the total separated in this stage. | 99.00 ±1.00% |
|  |  | Primary screening | Sorting screen | Controlled selection of different sizes of crushed inert, in two different channels (through conveyor belts): 30.00 ± 1.00% of particles greater than 20 mm at 3 inches called "coarse recycled aggregates" ready for storage, and 69.50 ± 1.00% of particles smaller than or equal to 20mm called "recycled fine aggregates" that will follow the following processes. In addition, a loss to the environment due to the generation of fine dust is considered, corresponding to 0.5 ± 0.5% of the total screened. | 30.00 ±1.00% of coarse aggregates and 69.50 ±1.00% of recycled fine aggregates. |
|  |  | Secondary crushing | Hammer mill | Crushing of inert fines smaller than or equal to 20mm to sizes approximately 1mm in average diameter. Considering a loss to the environment of dust, of 0.5 ± 0.5% of the total of the primary crushing. | 99.50 ±0.50% |
|  |  | Secondary screening | Screens from 10 to 2 mm | Controlled selection of different sizes of crushed fine inert, in 4 channels (through conveyor belts): 0.10 ± 0.10% of particles greater than 20 mm that could have passed through (coarse aggregates), 19.70 ± 1, 00% (approx. 1/5) of particles between 20mm to 10 mm, called "semi-fine aggregates", 39.40 ± 1.00% (approx. 2/5) of particles between 10mm to 1 mm, called "aggregates fine "and 39.40 ± 1.00% (approx. 2/5) of particles less than or equal to 1 mm, called" ultra-fine aggregates ". In addition, it is considered a loss to the environment due to the generation of fine powders, corresponding to 0.5 ± 0.5% of the total screened. | 0.10 ±0.10% of coarse aggregates, 19.70 ±1.00% of semi-fine aggregates, 39.40 ±1.00% of fine aggregates and 39.40 ±1.00% of ultra-fine aggregates. Approximate to the ratios of 1/5, 2/5, and 2/5, respectively. |
|  | Storing | Temporary storage of recycled coarse aggregates | - | Temporary storage of coarse aggregates in a designated place for it. Losses to the environment are not considered at this stage. | 100.00 ±0.00% |
|  |  | Temporary storage of recycled fine aggregates | - | Temporary storage of recycled fine aggregates at three different sites, designated for semi-fine, fine and ultra-fine aggregates. They are not considered losses to the environment at this stage. | 100.00 ±0.00% |
|  |  | Temporary storage of mined ferrous metals | - | Temporary storage of mined ferrous metals at a designated site. They are not considered losses to the environment at this stage. | 100.00 ±0.00% |
|  |  | Reject temporary storage | - | Temporary storage of the waste rejected in the selection of impurities from the inert entered into the plant, in a place designated for it. They are not considered losses to the environment at this stage. | 100.00 ±0.00% |
| Centralized bricks production plant | Secondary transport | Recycled coarse aggregates | Ffront loader and dump trucks with open hopper | Transportation of the recycled coarse aggregates to the place of sale. A loss to the environment due to loading, unloading and / or travel of 1.00 ± 1.00% of the total produced from this type of aggregate is considered. | 99.00 ±1.00% |
|  |  | Recycled semi-fine aggregates |  | Transportation of recycled semi-fine aggregates to the centralized block making plant. A loss to the environment due to loading, unloading and / or travel of 1.00 ± 1.00% of the total produced from this type of aggregate is considered. | 99.00 ±1.00% |
|  |  | Recycled fine aggregates |  | Transport of semi-recycled aggregates to the centralized block making plant. A loss to the environment due to loading, unloading and / or travel of 1.00 ± 1.00% of the total produced from this type of aggregate is considered. | 99.00 ±1.00% |
|  |  | Recycled ultra-fine aggregates |  | Transportation of recycled ultra-fine aggregates to the centralized block making plant. A loss to the environment due to loading, unloading and / or travel of 1.00 ± 1.00% of the total produced from this type of aggregate is considered. | 99.00 ±1.00% |
|  |  | Ferrous metals |  | Transport of the ferrous metals extracted to a transfer station that gives it a new use. They are not considered losses to the environment. | 100.00 ±0.00% |
|  |  | Rejects of selected aggregates |  | Transportation of the rejects of the selected inert to a municipal sanitary landfill. A loss to the environment due to loading, unloading and / or travel of 1.00 ± 1.00% of the total rejected is considered. | 99.00 ±1.00% |
|  | Post-recycling | Temporary storage of recycled fine aggregates | - | Temporary storage of recycled fine aggregates in three different sites, designated for semi-fine, fine and ultra-fine aggregates. They are not considered losses to the environment at this stage. | 100.00 ±0.00% |
|  |  | Mixing | Vertical shaft mixer | Mixing of the necessary inputs for the production of blocks, which will have these three types of fine aggregates as the main input. They are not considered losses to the environment at this stage. | 100.00 ±0.00% |
|  |  | Pressing | Block press | Pressing of the blocks, according to the established characteristics of dimensions, weight, and volume. It is considered a loss to the environment, to final disposal or possible to new recycling due to pressing failures or breakage of 5.00 ± 5.00% of the total of the manufactured blocks. | 95.00 ±5.00% |
|  |  | Storage and setting | Storage shelf | Storage of the manufactured blocks, allowing enough time for the hardening and loss of plasticity of the concrete block. They are not considered lost at this stage. | 100.00 ±0.00% |
|  |  | Sale of products | Shelf for displaying products for sale | Sale of the products obtained and available for sale, both for recycled coarse aggregates and for concrete blocks made with recycled fine aggregates. They are not considered lost at this stage. | 100.00 ±0.00% |
