# Supplementary material for: Construction and demolition waste recycling in developing cities: management and cost analysis
Source: Environ Sci Pollut Res Int. 2022 Nov 7;30(9):24377–97. doi: 10.1007/s11356-022-23502-x (PMC9938826; doi:10.1007/s11356-022-23502-x)
Supplement: Supplementary file 4 — Supplementary file4 (DOCX 16 KB) [file 11356_2022_23502_MOESM4_ESM.docx]

**Table S4:** Inventory of Scenario 2 (S2)

| **SITE OR PROCESSING STAGE** | **OPERATIONAL STAGE OF USE** | **PROCESSES INVOLVED** | **MACHINERY AND / OR EQUIPMENT** | **DETAILS** | **PERFORMANCE** |
| --- | --- | --- | --- | --- | --- |
| Generating source | Same of Scenario 1 | | | | |
| Fixed crushing plant | Same of Scenario 1 | | | | |
| Mobile crushing plant | Recycling | Transportation of mobile plant crushing plant | Mobile machinery | Transportation of all mobile machinery to the inert generation sites (mobile plants designed to process 42.86% of the total inert). For this reason, it is obviously not considered inert losses due to transport. | 100.00 ±0.00% |
|  |  | Collection of aggregates to be processed | Front loader | Inert reception that will be fed in the following processes. It is not considered lost. | 100.00 ±0.00% |
|  |  | Manual selection of recyclable aggregates and rejected aggregates | Low speed vibrating feeder | Selection of inert received, manual extraction of impurities considered as rejection corresponding to 5.00 ± 5.00% of the total of inert entered, approximately. | 95.00 ±5.00% |
|  |  | Pre-crushing | Pneumatic hammer | Reduction in the size of inert admitted greater than 30 cm in average diameter if they exist. Considering a loss to the environment due to the generation of dust, of 0.5 ± 0.5% of the total pre-crushed. | 99.50 ±0.50% |
|  |  | Trituration | Jaw crusher | Crushing of inert smaller than 30 cm to sizes approximately 20 mm in average diameter. Considering a loss to the environment due to the generation of dust, of 0.5 ± 0.5% of the total primary crushing. | 99.50 ±0.50% |
|  |  | Ferrous Metal Separation | Metal selection | extraction of steel chips from reinforced concrete waste. Considering an extraction of 1.00 ± 1.00% of the total separated in this stage. | 99.00 ±1.00% |
|  |  | Screening | Sorting screen | Controlled selection of crushed inert sizes with granulometries greater than and equal to 20 mm to 3 inches, called "recycled coarse aggregate" ready for storage. Considering a loss to the environment due to the generation of fine powders, corresponding to 0.5 ± 0 , 5% of the total screened. | 99.50 ±0.50% of coarse aggregates |
|  | Storage | Recycled coarse aggregates | - | Temporary storage of coarse aggregates in a designated place for it. They are not considered losses to the environment at this stage. | 100.00 ±0.00% |
|  |  | Ferrous metals | - | Temporary storage of mined ferrous metals at a designated site. They are not considered losses to the environment at this stage. | 100.00 ±0.00% |
|  |  | Rejects | - | Temporary storage of the waste rejected in the selection of impurities from the inert entered into the plant, in a place designated for it. They are not considered losses to the environment at this stage. | 100.00 ±0.00% |
| Centralized bricks production plant | Same of Scenario 1 | | | | |
