# Supplementary material for: Construction and demolition waste recycling in developing cities: management and cost analysis
Source: Environ Sci Pollut Res Int. 2022 Nov 7;30(9):24377–97. doi: 10.1007/s11356-022-23502-x (PMC9938826; doi:10.1007/s11356-022-23502-x)
Supplement: Supplementary file 5 — Supplementary file5 (DOCX 18 KB) [file 11356_2022_23502_MOESM5_ESM.docx]

**Table S5:** Inventory of Scenario 3 (S3)

| **SITE OR PROCESSING STAGE** | **OPERATIONAL STAGE OF USE** | **PROCESSES INVOLVED** | **MACHINERY AND / OR EQUIPMENT** | **DETAILS** | **PERFORMANCE** |
| --- | --- | --- | --- | --- | --- |
| Generation source | Separation and collection | Recovery and initial loss of generated RCDs | - | Recovery of RCD generated of 95.00%. Considering an inevitable loss to the environment of 5%. | 95.00 ±0.00% |
|  |  | Manual selection of hazardous and non-hazardous waste, at the source of generation | - | Selection of hazardous and non-hazardous waste generating sources (inert and non-inert), corresponding to an approximate at the municipal level of 0.10 ±0.03% and 99.90 ±14.37% respectively, of the total generated. | 99.90 ±14.37% |
| Fixed crushing plant | Primary transport | Primary transport of selected non-hazardous | Front loader and dump trucks with open hopper | Transportation of the total non-hazardous waste selected to the 5 fixed shredding plants, distributed equally to 20% of the total non-hazardous waste generated. Of the totals already transported, it is considering a loss to the environment due to loading, unloading and / or journey, of 1.00 ±1.00%. | 99.00 ±1.00% |
|  |  | Hazardous waste transportation | Front loader and dump trucks with open hopper | Transportation of hazardous waste to a municipal sanitary landfill, without considering a loss to the environment due to loading, unloading or journey. | 100.00 ±0.00% |
|  | Recycling | Collection of non-hazardous transported and received in plants | Vibrating pyramidal mailbox | Reception of non-hazardous that will be fed in the following processes. It is not considered lost. | 100.00 ±0.00% |
|  |  | Manual selection of inert, non-inert and rejects | Low speed vibrating feeder | Selection of non-hazardous received, inert and non-inert, corresponding to an approximate municipal level of 73.96 ±16.19% and 26.04 ±8.49%, respectively. These percentages are considered a loss at final disposal by manual selection of impurities considered as rejection corresponding to 5.00 ± 0.00%, both for inert and non-inert. Finally, the percentages discounting the loss are 68.96 ± 16.19% and 21.04 ±8.49%, respectively. | 68.96 ±16.19% |
|  |  | Pre-crushing | Pneumatic hammer | Reduction in the size of inert admitted greater than 30 cm in average diameter if they exist. Considering a loss to the environment due to the generation of dust, of 0.5 ±0.5% of the total pre-crushed. | 99.50 ±0.50% |
|  |  | Primary crushing | Jaw crusher | Crushing of inert smaller than 30 cm to sizes approximately 20 mm in average diameter. Considering a loss to the environment due to the generation of dust, of 0.5 ±0.5% of the total primary crushing. | 99.50 ±0.50% |
|  |  | Ferrous Metal Separation | Metal selection | extraction of steel chips from reinforced concrete waste. Considering an extraction of 1.00 ±1.00% of the total separated in this stage. | 99.00 ±1.00% |
|  |  | Primary screening | Sorting screen | Controlled selection of different sizes of crushed inert, in two different channels (through conveyor belts): 30.00 ±1.00% of particles greater than 20 mm at 3 inches called "coarse recycled aggregates" ready for storage, and 69.50 ±1.00% of particles smaller than or equal to 20 mm called "recycled fine aggregates" that will follow the following processes. In addition, a loss to the environment due to the generation of fine dust is considered, corresponding to 0.5 ±0.5% of the total screened. | 30.00 ±1.00% of coarse aggregates and 69.50 ±1.00% of recycled fine aggregates. |
|  |  | Secondary crushing | Hammer mill | Crushing of inert fines smaller than or equal to 20mm to sizes approximately 1mm in average diameter. Considering a loss to the environment of dust, of 0.5 ±0.5% of the total of the primary crushing. | 99.50 ±0.50% |
|  |  | Secondary screening | Screens from 10 to 2mm | Controlled selection of different sizes of crushed fine inert, in 4 channels (through conveyor belts): 0.10 ±0.10% of particles greater than 20 mm that could have passed through (coarse aggregates), 19.70 ±1, 00% (approx. 1/5) of particles between 20 mm to 10 mm, called "semi-fine aggregates", 39.40 ±1.00% (approx. 2/5) of particles between 10 mm to 1 mm, called "aggregates fine "and 39.40 ±1.00% (approx. 2/5) of particles less than or equal to 1 mm, called" ultra-fine aggregates". In addition, it is considered a loss to the environment due to the generation of fine powders, corresponding to 0.5 ±0.5% of the total screened. | 0.10 ±0.10% of coarse aggregates, 19.70 ± 1.00% of semi-fine aggregates, 39.40 ± 1.00% of fine aggregates and 39.40 ±1.00% of ultra-fine aggregates. Approximate to the ratios of 1/5, 2/5, and 2/5, respectively. |
|  | Storage | Recycled coarse aggregates | - | Temporary storage of coarse aggregates in a designated place for it. They are not considered losses to the environment at this stage. | 100,00±0,00% |
|  |  | Recycled fine aggregates | - | Temporary storage of recycled fine aggregates in three different sites, designated for semi-fine, fine and ultra-fine aggregates. They are not considered losses to the environment at this stage. | 100,00±0,00% |
|  |  | Selected non-inert and extracted ferrous metals | - | Temporary storage of selected non-inert and ferrous metals at two different designated sites. They are not considered losses to the environment at this stage. | 100,00±0,00% |
|  |  | Reject temporary storage | - | Temporary storage of the waste rejected in the selection of impurities from the inert entered into the plant, in a place designated for it. They are not considered losses to the environment at this stage. | 100,00±0,00% |
| Centralized bricks production plant | Same of Scenario 1 | | | | |
