# Supplementary material for: Construction and demolition waste recycling in developing cities: management and cost analysis
Source: Environ Sci Pollut Res Int. 2022 Nov 7;30(9):24377–97. doi: 10.1007/s11356-022-23502-x (PMC9938826; doi:10.1007/s11356-022-23502-x)
Supplement: Supplementary file 6 — Supplementary file6 (DOCX 19 KB) [file 11356_2022_23502_MOESM6_ESM.docx]

**Historical record of built and demolished areas of public order, Municipality of La Paz, 2013 – 2017.**

| Year | CONSTRUCTION m^2^ year^-1^ | | | | | | | | | | | | | | | | | REFURBISHMENT AND  MAINTENANCE  m^2^ year^-1^ | DEMOLITION + OPENING  m^2^ year-^1^ | Mov. LANDS  m^3^ year^-1^ |
| --- | --- | --- | --- | --- | --- | --- | --- | --- | --- | --- | --- | --- | --- | --- | --- | --- | --- | --- | --- | --- |
|  | BUILDING WORKS | | | | | | CIVIL WORKS OF PAVED AREAS | | | | | CIVIL WORKS OF WATER SYSTEMS | | CIVIL WORKS OF DRAINS | CIVIL WORKS OF FENCES | | |  |  |  |
|  | COMMUNAL HOUSES | MARKETS | POLICE  MODULES | HYGIENIC SERVICES | PLATFORMS | HEALTH CENTERS | SIDEWALK CORDS | SURFACE  AREAS | VEHICULAR BRIDGES | STANDS | PEDESTRIAN BRIDGES | RIDGED | CANALS | DRAINS | BREAKWATERS | FENCE  WALL | CONTENTION WALL |  |  |  |
| 2013 | 12000 | 58500 | 9000 | 1950 | 8000 | 0 | 40132 | 2757 | 0 | 9877 | 7800 | 51000 | 5500 | 11700 | 6500 | 112021 | 4249 | 2738 | 41956 | 3899 |
| 2014 | 13500 | 13500 | 4500 | 0 | 22000 | 7500 | 32690 | 6125,5 | 0 | 10476 | 2600 | 58000 | 3250 | 14950 | 7500 | 57888 | 3948 | 1661 | 45577 | 2138 |
| 2015 | 7500 | 13500 | 1500 | 650 | 2000 | 7500 | 27331 | 11596 | 0 | 15772 | 3250 | 19000 | 500 | 1950 | 1500 | 37012 | 2246 | 769 | 24156 | 1471 |
| 2016 | 13500 | 9000 | 1500 | 5200 | 0 | 0 | 38543 | 2221,41 | 2000 | 4426 | 1300 | 30000 | 1500 | 16250 | 2000 | 49811 | 2123 | 1115 | 18400 | 2443 |
| 2017 | Sd | Sd | Sd | Sd | Sd | Sd | Sd | Sd | Sd | Sd | Sd | Sd | Sd | Sd | Sd | 61967 | 1097 | 633 | 12881 | 2099 |

*Note: Sd = no data*

*Source: Own elaboration, based on the report of CDW management of the city of La Paz, 2018*

**Historical record of built and demolished areas of the private order, Municipality of La Paz, 2013 – 2017.**

| YEARS | CONSTRUCTION m^2^ year^-1^ | | | REFURBISHMENT AND MAINTENANCE m^2^ ^year-1^ | DEMOLITION + OPENING  m^2^ ^year-1^ | Mov. LANDS  m^3^ ^year-1^ |
| --- | --- | --- | --- | --- | --- | --- |
|  | BUILDING WORKS | CIVIL WORKS | |  |  |  |
|  | PRIVATE BUILDINGS | FENCE WALL | CONTENTION WALL |  |  |  |
| 2013 | 541772 | 10244 | 9400 | 6600 | 101152 | 270072 |
| 2014 | 873680 | 17545 | 9500 | 7380 | 202552 | 257264 |
| 2015 | 841270 | 16641 | 10900 | 5700 | 178966 | 274213 |
| 2016 | 761524 | 12685 | 14600 | 6660 | 109948 | 297643 |
| 2017 | 767937 | 9042 | 17300 | 5220 | 106162 | 510728 |

*Source: Own elaboration, based on the report of CDW management of the city of La Paz, 2018*
