# Supplementary material for: Construction and demolition waste recycling in developing cities: management and cost analysis
Source: Environ Sci Pollut Res Int. 2022 Nov 7;30(9):24377–97. doi: 10.1007/s11356-022-23502-x (PMC9938826; doi:10.1007/s11356-022-23502-x)
Supplement: Supplementary file 7 — Supplementary file7 (DOCX 24 KB) [file 11356_2022_23502_MOESM7_ESM.docx]

**Table S6**: Construction and demolition waste (CDW) characterization.

| **N.** | **References** | | **City, region, or country** | | **Construction waste [%]** | | | | | | | | | | | | | | | | | | | | | | | | | | | | | | | | | |
| --- | --- | --- | --- | --- | --- | --- | --- | --- | --- | --- | --- | --- | --- | --- | --- | --- | --- | --- | --- | --- | --- | --- | --- | --- | --- | --- | --- | --- | --- | --- | --- | --- | --- | --- | --- | --- | --- | --- |
|  |  |  |  |  | Concrete | | | Masonry | | Bricks | | Bricks and masonry | | Sand | | Gypsum | | Ceramics | Timber | | | Metals | Plastics | | Glass | | Paper and cardboard | Hazardous | | | Mixed CDW | | | Others | | | TOTAL | |
| 3 | Lu et al. (2011) | | Shenzhen, China | | 22.72 | | | 6.09 | | 7.11 | |  | |  | |  | |  | 29.74 | | | 0.73 | 0.58 | |  | |  |  | | |  | | | 33.03 | | | 100 | |
| 20 | Myhre (2000) | | Norway | |  | | |  | |  | | 33.85 | |  | | 4.17 | |  | 14.58 | | | 1.04 |  | |  | |  | 0.52 | | | 45.83 | | |  | | | 100 | |
|  |  |  |  |  |  | | |  | |  | | 36.94 | |  | | 8.24 | |  | 25.88 | | | 2.82 |  | | 0.71 | |  | 2.82 | | | 22.59 | | |  | | | 100 | |
| 22 | Cochran et al. (2007) | | Florida, USA | | 69.33 | | |  | |  | |  | |  | | 10.92 | |  | 6.93 | | | 2.94 |  | |  | |  |  | | | 9.87 | | |  | | | 100 | |
| 26 | Mah et al. (2016) | | Malaysia | | 40 | | | 12 | | 7 | |  | | 13 | | 1 | | 5 | 13 | | | 8 |  | |  | |  |  | | |  | | |  | | | 100 | |
|  |  |  |  |  | 39 | | | 10 | | 10 | |  | | 19,5 | | 1 | | 8 | 7 | | | 5,5 |  | |  | |  |  | | |  | | |  | | | 100 | |
| 28 | Mercader y Ramírez (2013) | | Sevilla, Spain | | 85.13 | | |  | | 10.48 | |  | |  | | 0.85 | |  | 0.55 | | | 0.77 | 0.77 | |  | | 0.86 |  | | |  | | | 0.59 | | | 100 | |
| 29 | Kofoworola y Gheewala (2009) | | Thailand | |  | | |  | |  | | 45.84 | |  | | 6.25 | |  | 13.68 | | | 1.32 | 4.51 | | 0.46 | |  | 2.10 | | |  | | | 25.84 | | | 100 | |
| **N.** | **References** | | | **City, region, or country** | | **Demolition waste [%]** | | | | | | | | | | | | | | | | | | | | | | | | | | | | | | | |  |
|  |  |  |  |  |  | Concrete | Masonry | | Bricks | | Bricks and masonry | | Sand | | Gypsum | | Ceramics | | | Timber | Metals | | Plastics | Glass | | Paper and cardboard | | | Hazardous | Mixed CDW | | | Others | | | TOTAL | |  |
| 1 | Ram y Kalidindi (2017) | | | Chennai, India | | -----------------------76.00----------------------- | | | | | | |  | | | | | | | 19.00 | | |  | | | | | | | | | | | | | 95 | |  |
| 17 | Srour et al. (2013) | | | Beirut, Lebanon | | 87 |  | | | | | | | | | | | | | | | | | | | | | | | | | | | | | 87 | |  |
| 22 | Cochran et al. (2007) | | | Florida, USA | | 81.62 |  | |  | |  | |  | |  | |  | | | 0.18 | 5.2 | |  |  | |  | | |  | 13 | | |  | | | 100 | |  |
| 24 | Metro Vancouver (2008) | | | Canada | | 25 |  | |  | |  | |  | | 2 | |  | | | 44 | 3 | |  |  | |  | | |  | 26 | | |  | | | 100 | |  |
| 26 | Mah et al. (2016) | | | Malasia | | 20 | 9 | | 9 | |  | |  | |  | |  | | |  | 62 | |  |  | |  | | |  |  | | |  | | | 100 | |  |
| **N.** | | **References** | | **City, region, or country** | | **Mixed CDW [%]** | | | | | | | | | | | | | | | | | | | | | | | | | | | | | | | |  |
|  |  |  |  |  |  | Concrete | Masonry | | Bricks | | Bricks and masonry | | Sand | | Gypsum | | Ceramics | | | Timber | Metals | | Plastics | Glass | | Paper and cardboard | | | Hazardous | Mixed CDW | | Others | | | TOTAL | | |  |
| *8* | | Bergsdal et al. (2007) | | Norway | | 67.24 | | | | | | |  | |  | |  | | | 14.58 | 3.63 | |  |  | |  | | |  |  | | 14.55 | | | 100 | | |  |
| *18* | | Mañá et al. (2000) | | España | | 85 | | | | | | |  | |  | |  | | | 11.2 | 1.8 | | 0.2 |  | |  | | |  |  | | 1.8 | | | 100 | | |  |
| *30* | | Pereira (2002) | | Portugal | | 58.3 | | | | | | |  | |  | |  | | | 8.3 | 8.3 | | 0.83 |  | |  | | |  |  | | 24.27 | | | 100 | | |  |
| *31* | | Costa y Ursella (2003) | | Italia | | 84.3 | | | | | | |  | |  | |  | | |  | 0.08 | |  |  | |  | | |  |  | | 15.62 | | | 100 | | |  |
