# Supplementary figures and images for: Construction and demolition waste recycling in developing cities: management and cost analysis
Source: Environ Sci Pollut Res Int. 2022 Nov 7;30(9):24377–97. doi: 10.1007/s11356-022-23502-x (PMC9938826; doi:10.1007/s11356-022-23502-x)

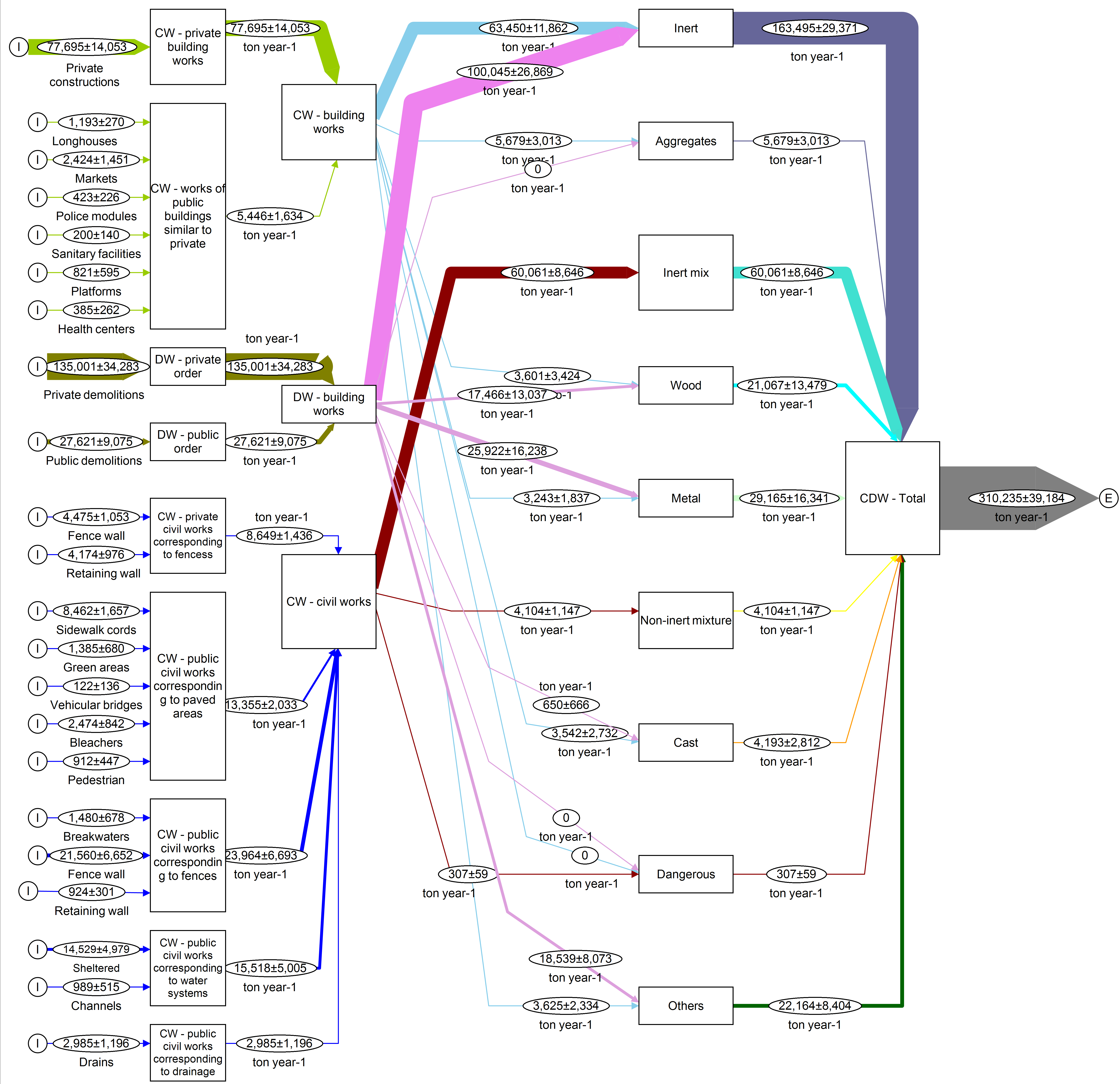

Supplement: Supplementary file 8 — Supplementary file8 (PNG 1173 KB) [file 11356_2022_23502_MOESM8_ESM.png]

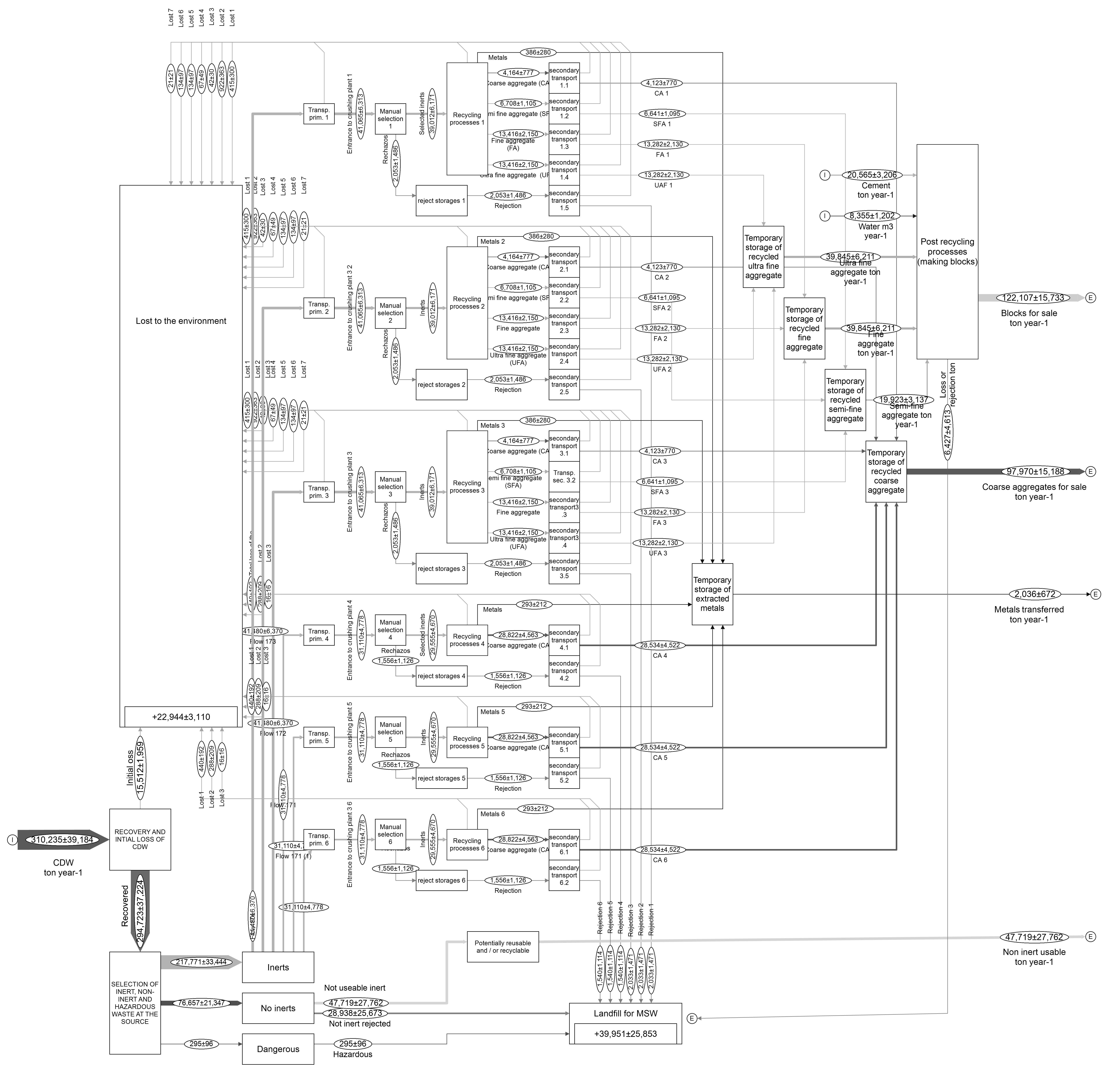

Supplement: Supplementary file 9 — Supplementary file9 (JPG 3085 KB) [file 11356_2022_23502_MOESM9_ESM.jpg]

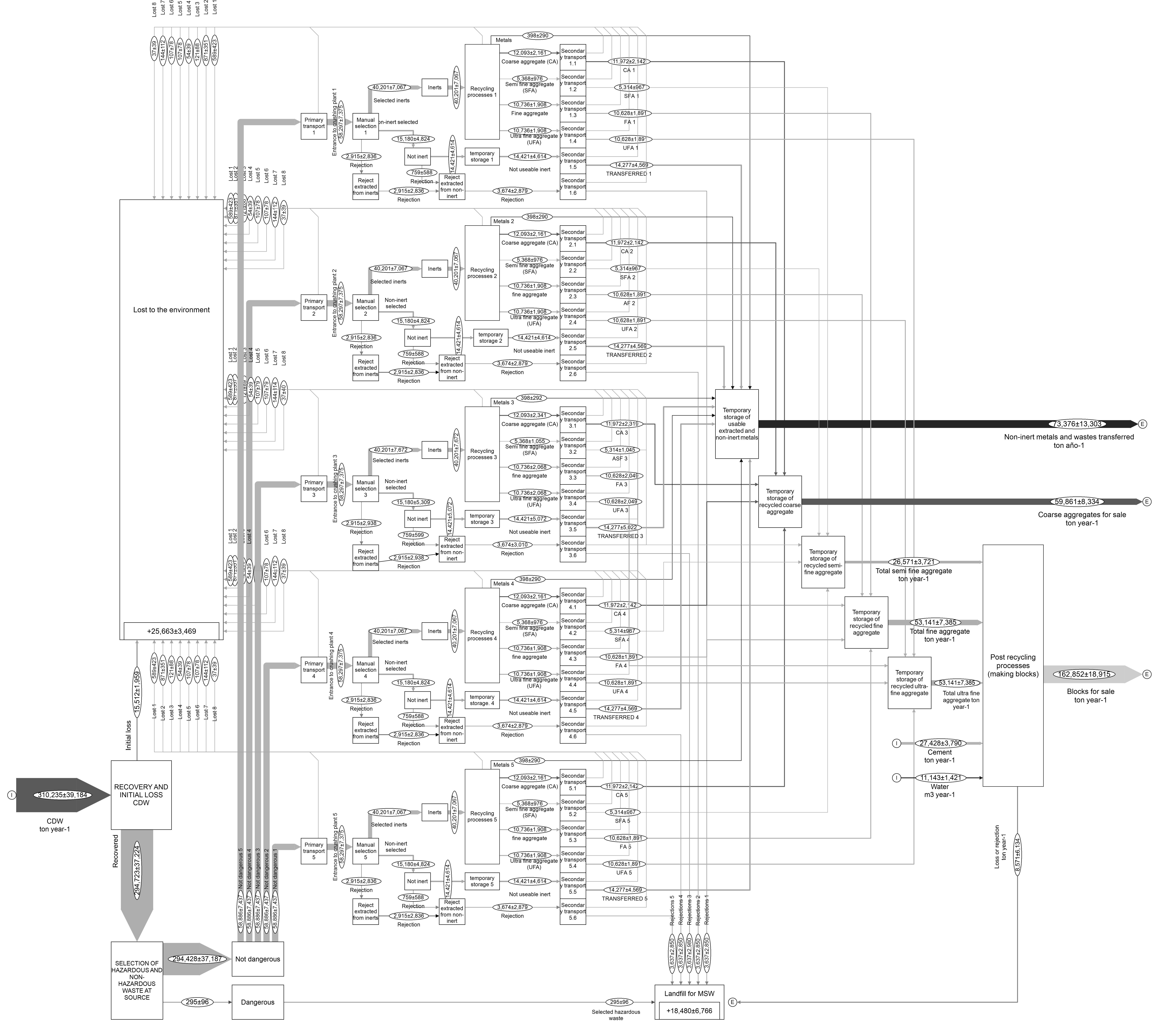

Supplement: Supplementary file 10 — Supplementary file10 (JPG 4424 KB) [file 11356_2022_23502_MOESM10_ESM.jpg]

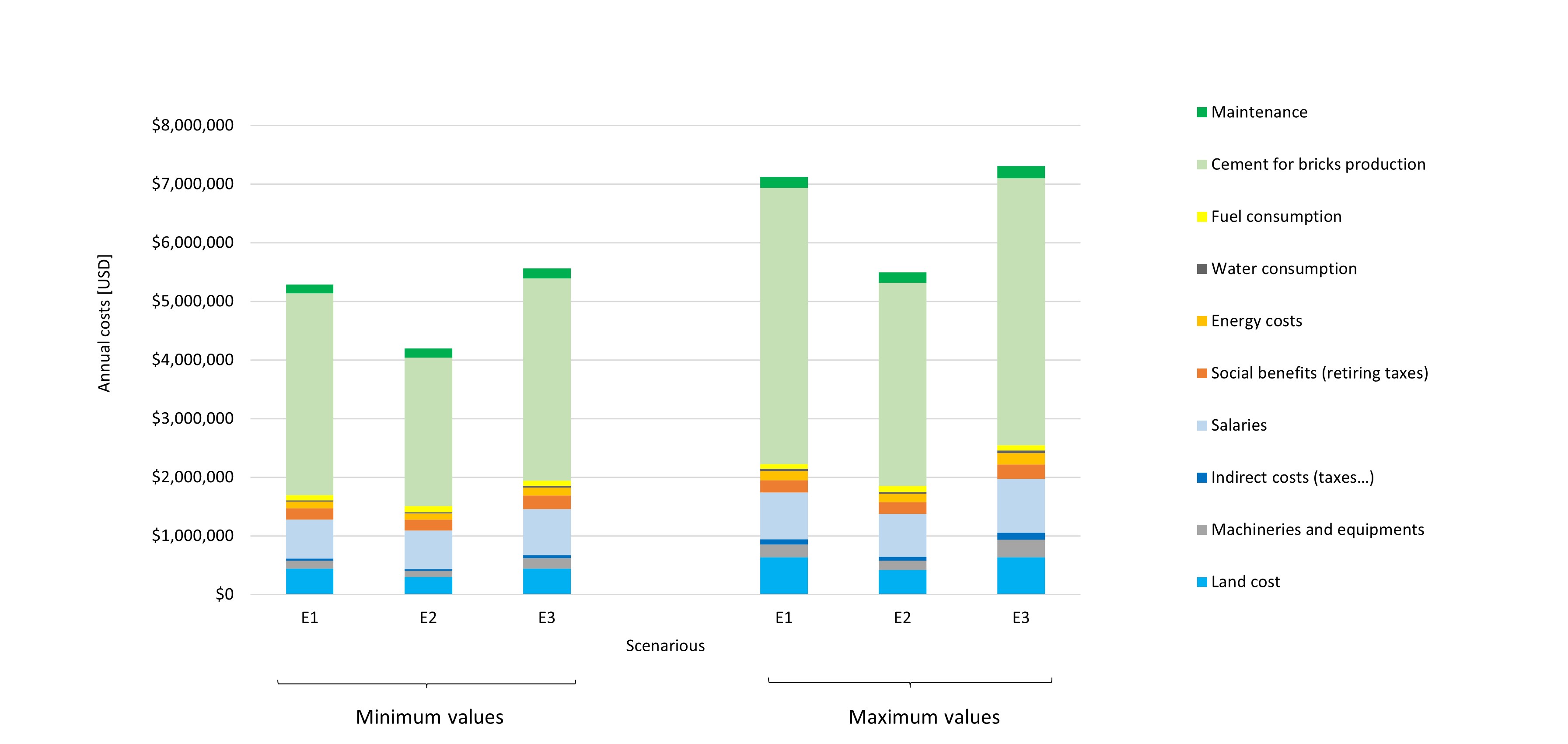

Supplement: Supplementary file 11 — Supplementary file11 (JPG 280 KB) [file 11356_2022_23502_MOESM11_ESM.jpg]
